# Anti-TNFα and Anti-IL-1β Monoclonal Antibodies Preserve BV-2 Microglial Homeostasis Under Hypoxia by Mitigating Inflammatory Reactivity and ATF4/MAPK-Mediated Apoptosis

**DOI:** 10.3390/antiox14030363

**Published:** 2025-03-19

**Authors:** Linglin Zhang, Chaoqiang Guan, Sudena Wang, Norbert Pfeiffer, Franz H. Grus

**Affiliations:** 1 Department of Ophthalmology, University Medical Center, Johannes Gutenberg University Mainz, 55131 Mainz, Germany; lzhang01@uni-mainz.de (L.Z.); cguan@uni-mainz.de (C.G.); norbert.pfeiffer@unimedizin-mainz.de (N.P.); 2 Department of Anesthesiology, University Medical Center, Johannes Gutenberg University Mainz, 55131 Mainz, Germany; suwang@uni-mainz.de

**Keywords:** microglia, hypoxia, mitochondria, inflammatory pathways, anti-TNFα, anti-IL-1β, proteomics, apoptosis

## Abstract

The disruption of microglial homeostasis and cytokine release are critical for neuroinflammation post-injury and strongly implicated in retinal neurodegenerative diseases like glaucoma. This study examines microglial responses to chemical hypoxia induced by cobalt chloride (CoCl_2_) in BV-2 murine microglial cells, focusing on signaling pathways and proteomic alterations. We assessed the protective effects of monoclonal antibodies against TNFα and IL-1β. CoCl_2_ exposure led to decreased cell viability, reduced mitochondrial membrane potential, increased lactate dehydrogenase release, elevated reactive oxygen species generation, and activation of inflammatory pathways, including nitric oxide synthase (iNOS), STAT1, and NF-κB/NLRP3. These responses were significantly mitigated by treatment with anti-TNFα and anti-IL-1β, suggesting their dual role in reducing microglial damage and inhibiting inflammatory reactivity. Additionally, these treatments reduced apoptosis by modulating ATF4 and the p38 MAPK/caspase-3 pathways. Label-free quantitative mass spectrometry-based proteomics and Gene Ontology revealed that CoCl_2_ exposure led to the upregulation of proteins primarily involved in endoplasmic reticulum and catabolic processes, while downregulated proteins are associated with biosynthesis. Anti-TNFα and anti-IL-1β treatments partially restored the proteomic profile toward normalcy, with network analysis identifying heat shock protein family A member 8 (HSPA8) as a central mediator in recovery. These findings offer insights into the pathogenesis of hypoxic microglial impairment and suggest potential therapeutic targets.

## 1. Introduction

Reduced oxygen supply is a common stressor that induces cell damage during the initiation and progression of retinal neurodegenerative disease [1]. Among these, glaucoma is the leading cause of irreversible visual disability [2], characterized by the progressive death of retinal ganglion cells (RGCs). In glaucoma, retinal hypoxia arises from impaired local blood flow due to elevated IOP or vascular dysregulation [3]. The retina, with its high metabolic demand, is particularly susceptible to hypoxia, which triggers inflammation, oxidative stress, and mitochondrial dysfunction [4]. However, the detailed mechanisms underlying hypoxia-induced retinal injury are extremely complicated and remain unclear.

Microglia are the resident macrophages and serve as a major component of the immune system within the retina, regulating the local microenvironment and participating in the modulation of synaptic plasticity [5]. Disruption of microglia homeostasis represents a common pathomechanism in a variety of retinal degenerative diseases and often occurs concurrently with or precedes overt RGC death [6]. Injury signals rapidly activate microglia reactivity, triggering migration, enhanced phagocytosis, and release of neurotoxins and inflammatory cytokines or chemokines, which exacerbate retina damage and promote pro-apoptotic events [6]. Notably, among the cytokines and chemokines induced by hypoxia, tumor necrosis factor α (TNFα) and interleukin-1 beta (IL-1β) exhibit the most significant upregulation [7]. They act as inflammation amplifiers and trigger self-enhancing inflammatory cascades, and promote microglia activation in a positive feedback mechanism [8,9]. This enhanced inflammation causes secondary RGC death [10], reactive gliosis [11], and disruption of the blood–retinal barrier [12]. Growing evidence from rodent studies suggests that excessive release of TNF-α and IL-1β by microglia exerts detrimental effects on RGCs [10,13]. Therefore, targeting these cytokines released by microglia could be a potential pharmacological strategy for the treatment of retinal neurodegeneration.

Monoclonal antibodies, neutralizing TNFα and IL-1β (anti-TNFα and anti-IL-1β), emerged as a significant treatment option for central nervous system (CNS) autoimmune diseases [14,15]. The potential protective effects of anti-TNFα and anti-IL-1β in neurodegenerative disorders have gained increased attention over the past decade. One clinical retrospective cohort study reported that early exposure to anti-TNFα therapy was associated with substantially reduced Parkinson’s disease incidence [16]. In addition, it was reported that systemically administered anti-IL-1β exerts neuroprotective effects on injury in animal fetal brains against hypoxic–ischemic reperfusion (I/R)-related injury [17]. However, mechanisms underlying the neuroprotective effects of anti-TNFα and anti-IL-1β remain largely unclear.

A well-controlled and simplified in vitro system can serve as a reliable approach to explore the effects of anti-TNFα and anti-IL-1β specifically on hypoxia-injured microglia and their exact molecular mechanisms. Using cobalt chloride (CoCl_2_), a widely used hypoxia-mimetic agent, we aimed to determine whether and how anti-TNFα and anti-IL-1β exert protective effects on BV-2 microglia against hypoxic stress, in terms of cell viability, oxidative stress, and apoptosis. Following this, we examined the anti-inflammatory effects of anti-TNFα and anti-IL-1β on microglial polarization and inflammatory pathways under hypoxia. Furthermore, proteome changes were analyzed via a label-free mass spectrometry (MS)-based quantitative proteomics approach. This study provides a novel understanding of the molecular mechanisms involved in the microglial response to hypoxic stress and determines the hypothesis that hypoxia-injured microglia can be rescued by anti-TNFα and anti-IL-1β.

## 2. Materials and Methods

1. Chemicals and reagents

CoCl_2_, paraformaldehyde, Dulbecco’s Phosphate-Buffered Saline (DPBS), Triton X-100 solution, and normal donkey serum (NDS) were purchased from Sigma-Aldrich (St. Louis, MO, USA). RPMI 1640 Medium was acquired from Pan-biotech (Bayern, Germany). Fetal bovine serum was provided by (FBS; Cambrex Bioscience, Verviers, Belgium). L-alanyl-L-glutamin was obtained from Bio&SELL (Feucht, Germany). Penicillin/streptomycin solution, RIPA lysate, Lactate dehydrogenase (LDH) detection kit, Diamidinyl phenyl indole staining (DAPI), Protease and Phosphatase Inhibitor Cocktail, BCA protein quantitative kit, and NuPAGE 4–12% Bis-Tris gels were procured from Thermo Fisher Scientific (MA, USA). MTS Assay was purchased from Promega (Walldorf, Germany). DCFDA intracellular reactive oxygen species (ROS) assay kit and Phalloidin-iFluor 488 Reagent were obtained from Abcam (Cambridge, UK). JC-10 assay was supplied by G-Biosciences (Saint Louis, MO, USA). The TUNEL assay kit was obtained from Roche-Applied-Science (Mannheim, Germany). SignalFire™ ECL reagent was purchased from Cell Signaling Technology (MA, USA). Appendix A shows the information of the therapeutic antibodies, primary antibodies, and secondary antibodies involved in this study.

2. BV-2 cell line cultivation

The immortalized murine microglial BV-2 cell line (Interlab Cell Line Collection [18]) was a generous gift from Prof. Jürgen Winkler (University of Erlangen—Nuremberg, Germany). The BV-2 cells were cultured in RPMI 1640 medium with 10% FBS, 1% L-alanyl-L-glutamine, and 1% penicillin/streptomycin in T75 flasks. Cells were maintained at 37 °C in a humidified 5% CO_2_ environment.

3. Establishment of hypoxic model in vitro

CoCl_2_ was used to simulate chemical hypoxic conditions in BV-2 cells. The cells were seeded (9 × 104 cells/mL) in 96-well plates (100 µL/well) and allowed to grow for 24 h. With good water solubility, different concentrations of CoCl_2_ solution were diluted by serum-free culture medium. The cells were exposed to CoCl_2_ at concentrations ranging from 10 to 120 µM for 24 h. A concentration that moderately inhibited cell viability (about 50%) was selected for subsequent experiments.

4. Fluorescein–phalloidin staining

The effects of CoCl_2_-induced hypoxia on BV-2 cell morphology were assessed with fluorescein–phalloidin staining of cells’ actin cytoskeleton. After CoCl_2_ stress, cells were fixed with 4% paraformaldehyde for 10 min at RT, washed twice with PBS, and permeabilized in 0.1% Triton X-100 for 5 min. Subsequently, the cells were incubated with 100 µL phalloidin (1:200) for actin filament labeling at room temperature for 60 min. After rinsing twice with PBS, the cells were stained with DAPI (1:1000) for 10 min to label the nucleus, followed by rinsing three times. Images were captured by fluorescence microscopy (Eclipse TS 100; Nikon, Tokyo, Japan).

5. MTS assay

Viable cells can reduce MTS tetrazolium compounds to generate colored formazan dye. After treatments, cells were incubated with CellTiter 96^®^ AQueous One Solution Reagent (20 µL of reagent in a final volume of 120 µL of culture medium in the 96-well plate) at 37 °C for 1 h. Cells were treated with neither CoCl_2_ nor antibodies served as normal control with 100% cell viability. Blank wells were set by adding 20 µL of reagent to the final volume of 120 µL of the medium without any cells. The absorbance was measured at 450 nm using a microplate reader (Multiskan Ascent, Thermo Labsystems, Waltham, MA, USA) to quantitatively assess the generation of colored formazan, which is proportional to the amount of metabolically active cells. Cell viability was calculated according to the following formula:

viability (%)  =  OD (measured value)  −  OD (blank value)/OD (control value)  −  OD (blank value) × 100%

6. Cell treatment

Anti-TNFα and anti-IL-1β were freshly diluted in cell culture medium. To determine the safe concentration ranges of the antibodies, BV-2 cells were treated with anti-TNFα or anti-IL-1β at concentrations ranging from 1 μg/mL to 16 μg/mL for 24 h. Cell viability was determined by MTS assay as described above.

Then, we evaluated the effects of anti-TNFα or anti-IL-1β on BV-2 cells under hypoxia. Following CoCl_2_-induced hypoxic injury, cells were treated with anti-TNFα or anti-IL-1β at concentrations within the safe ranges for another 24 h. Cell viability was measured by MTS assay. The optimal concentrations of anti-TNFα and anti-IL-1β were determined based on the highest cell viability and applied in subsequent experiments. Then, cells were divided into four groups: normal control cells, hypoxia model cells (CoCl_2_), anti-TNFα-treated cells (CoCl_2_ + anti-TNFα), and anti- IL-1β-treated cells (CoCl_2_ + anti- IL-1β).

7. Lactate dehydrogenase (LDH) assay

The LDH assay was performed using CyQUANT™ LDH Cytotoxicity Assay following the manufacturer’s protocols. BV-2 cells were cultured overnight. For spontaneous LDH activity control, 10 μL of sterile ultrapure water was added to one set of triplicate wells of cells. For the experiment, the cells were treated as described above. Then, on the day of the experiment, 10 μL of 10×Lysis Buffer was added to the set of triplicate wells of cells serving as the Maximum LDH activity controls. The plate was incubated at 37 °C for 45 min. After that, 50 µL of each well’s supernatant was transferred to a new 96-well plate, followed by adding 50 µL of LDH reaction solution to each well and 30 min of room-temperature incubation protected from light. Lastly, absorbance values were determined at 490 nm wavelength with values of the background subtracted. The cytotoxicity rate was calculated using the following formula:

Cytotoxicity (%) = (Experiment treated LDH activity − Spontaneous LDH activity control)/(Maximum LDH activity control − Spontaneous LDH activity control) × 100.

8. Intracellular ROS measurement

The ROS generation was determined using the fluorogenic probe DCFH-DA-Cellular ROS assay kit according to the manufacturer’s instructions. Briefly, DCFH-DA was diluted with a serum-free medium at 1:1000 to a final concentration of 10 μM. BV-2 cells were incubated with diluted DCFH-DA dye for 45 min in the dark. After removing the dye solution, the cells were treated with 100 µL/well of culture medium containing CoCl_2_, with or without anti-TNFα or anti-IL-1β, at 37 °C in 5% CO_2_ for 2 h. The emitted fluorescence intensity was measured using a fluorescence microplate reader (Fluoroskan Ascent FL, Thermo Labsystems, MA, USA) at Ex/Em = 485/535 nm. Blank readings were subtracted from all measurements. Fold changes were determined from the control group. Representative images were taken under a fluorescence microscope (Eclipse TS 100; Nikon, Tokyo, Japan).

9. Mitochondrial membrane potential (MMP) assessment, JC-10

The MMP was determined using the JC-10 fluorescence quantitative assay. JC-10 becomes concentrated to form red fluorescent aggregates in healthy mitochondria and reversibly converts red fluorescence into green fluorescence when the membrane potential collapses. When the MMP collapses, the JC-10 dye is not retained and the dye reverts to its monomeric green form in the cytoplasm. For the assay, BV-2 cells were treated as described above and stained with the JC-10 dye (15 μM) for 25 min at 37 °C in the dark. Then, the samples were washed 3 times with PBS. The intensity of red and green fluorescence was recorded by a fluorometer microplate reader at Ex/Em = 530/590 nm and Ex/Em = 485/535 nm, respectively. Representative images were taken under a fluorescence microscope (Eclipse TS 100; Nikon, Tokyo, Japan).

10. Immunofluorescence staining

After the treatment described above, BV-2 cells were fixed with 4% paraformaldehyde for 10 min, then permeabilized and blocked with 0.1% Triton X-100 and 5% NDS for 1 h. Subsequently, the cells were incubated with primary antibody anti-iNOS (1:200) at 4 °C overnight, and with Alexa Fluor 488-conjugated secondary antibody (1:200) for 1 h at RT. Cell nuclei were labeled with DAPI (1:1000) for 10 min. After washing, cells were examined by a fluorescence microscope (Eclipse TS 100; Nikon, Tokyo, Japan).

11. TUNEL assay

TUNEL assay was performed to detect apoptotic cells according to the manufacturer’s protocol. Briefly, after fixation, cells were permeabilized with 0.1% Triton X-100 for 2 min on ice and rinsed three times with PBS for 5 min. An amount of 50 μL TUNEL reaction mixture was added to each sample and incubated for 1 h at 37 °C in the dark. DAPI was used for counterstaining. Cells were washed with PBS and then observed using fluorescence microscopy (Eclipse TS 100; Nikon, Tokyo, Japan). At least 400 cells were observed per experimental group.

12. Western blot analysis

After treatment, BV-2 cells were washed twice with cold PBS. Total protein was extracted using RIPA buffer containing a 1% Protease and Phosphatase Inhibitor Cocktail. The protein concentration was then determined using the BCA protein assay kit. Equal amounts of protein (30~40 µg) per lane were separated on NuPAGE 4–12% Bis-Tris gels, followed by transferring to a 0.45 µm PVDF membrane. Then, membranes were blocked with 5% bovine serum albumin in 1 × Tris-buffered saline containing 0.1% Tween 20 (TBST) for 1 h. Then, membranes were incubated with diluted primary antibodies (1:1000) in TBST overnight at 4 °C. After washing with TBST thrice, membranes were incubated with HRP-conjugated secondary antibodies diluted with TBST (1:8000) for 1 h at room temperature. The membranes were then washed thrice and developed with SignalFire™ ECL reagent according to the manufacturer’s instructions. Visual detection was performed using the Fluor Chem E system (ProteinSimple, San Jose, CA, USA). The densitometric analysis was performed using ImageJ (version 1.54g) and β-actin was used as a reference protein to normalize the target proteins.

13. Liquid chromatography–mass spectrometry (LC–MS/MS)-based proteomics analysis

Protein extraction and concentration determination were performed as described above. The LC–MS/MS measurements were carried out using the Hybrid Linear Ion Trap-Orbitrap MS system (LTQ Orbitrap XL; Thermo Scientific, Bremen, Germany), equipped with the EASY-nLC 1200 system (Thermo Scientific, Bremen, Germany). The aqueous solvent A consisted of 0.1% formic acid in LC-MS-grade water and the organic solvent B consisted of 0.1% formic acid in acetonitrile. After trypsin digestion and peptide purification as Perumal et al. described [19], samples were dissolved in 80 µL of solvent A and 2 µL of each sample was injected into the system for each run. The peptides were eluted with gradient solvent B (0.1% formic acid in acetonitrile). The settings and MS parameters for the analysis are listed in detail [19]. Continuum mass spectra data were acquired on the ESI-LTQ-Orbitrap-XL MS (Thermo Scientific, Bremen, Germany). Raw LC-MS data were processed using the MaxQuant computational proteomics platform (v.1.6.1.0) for peptide and protein identification. The tandem MS spectra were searched against UniProt databases for Mus musculus and Homo sapiens with the standard settings as described in a previous study [19]. A false discovery rate (FDR) cutoff of 1% was used to filter protein identifications. The MaxQuant output file was processed with Perseus software (version 1.6.2.3). Protein LFQ intensities were log2-transformed and missing values were assigned random values using the imputation principle (downshift 1.8, width 0.3, total matrix mode). Proteins with differential expression levels among the four groups (Ctrl, CoCl_2_, CoCl_2_ + anti-TNFα, CoCl_2_ + anti-IL-1β) were initially screened in batches. Heatmap and volcano plots were generated. Protein–protein interactions were analyzed using the String database (https://string-db.org/, accessed on 16 December 2024).

14. Scratch wound migration assay

For the scratch wound assay, 300,000 BV-2 cells were seeded into each well in 6-well plates (Fisher Scientific). After 24 h of growth, the cells reached approximately 80% confluent. The monolayer was scratched with a sterile 200 μL pipette tip, and the cells were gently washed twice with PBS. The cells were allowed to recover in a serum-free medium containing CoCl_2_, with or without anti-TNFα or anti-IL-1β. The wound closure was then viewed after 12 h and 24 h under the microscope (Eclipse TS 100; Nikon, Tokyo, Japan).

15. Statistical Analysis

All data are presented as mean ± standard deviation (SD). The normality of the data distribution was evaluated using the Shapiro–Wilk test. One-way Analysis of Variance (ANOVA) followed by Tukey’s post hoc test was used for multiple comparisons. The Brown–Forsythe test was employed to verify the assumption of equal variances in ANOVA. Post hoc tests were conducted only if the F-test resulted in *p* < 0.05, and no significant variance inhomogeneity was detected among the groups. Student’s t-test was used for two-way comparisons. Statistical analyses were performed using GraphPad Prism 10.0, and differences were considered statistically significant at *p* < 0.05.

## 3. Results

### 3.1. Anti-TNFα and Anti-IL-1β Increased Cell Viability and Reduced Cytotoxicity in CoCl_2_-Treated BV-2 Cells

**Figure 1:**
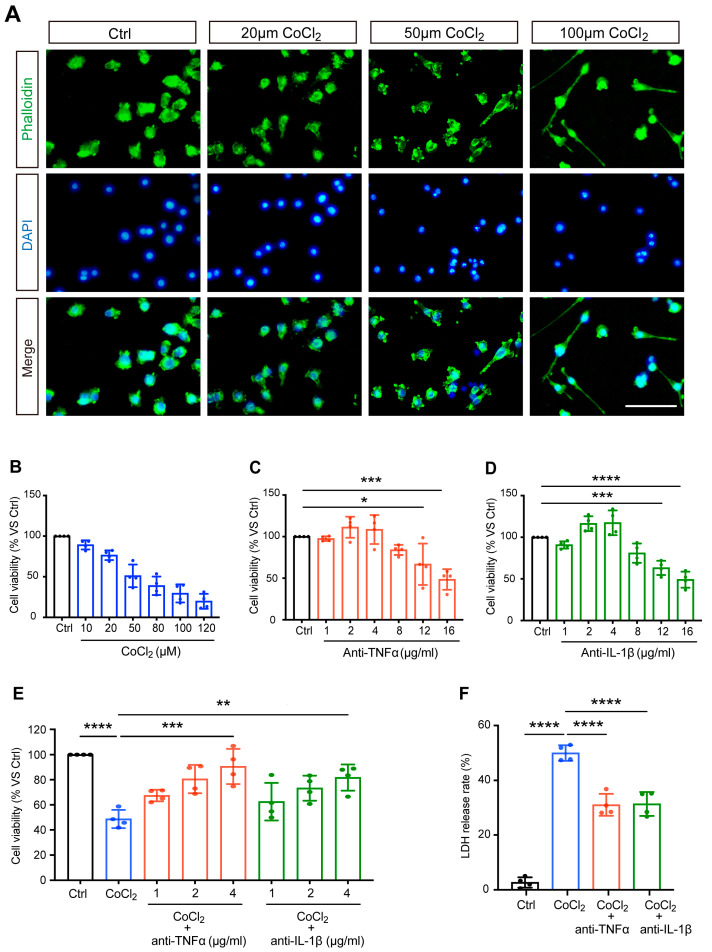
Anti-TNFα and anti-IL-1β treatments reduced CoCl_2_-induced loss of BV-2 cell viability and LDH release. (**A**) The impact of CoCl_2_ on cellular viability and morphology of BV-2 cells. Cells were treated with the indicated concentration of CoCl_2_ for 24 h. Representative images of BV-2 cells immunostained with phalloidin for F-actin (green) and DAPI for nuclei (blue) detection (scale bar = 50 μm). (**B**–**D**) The cell viability of BV-2 cells upon exposure to increasing CoCl_2_ concentrations (from 10 to 120 µM), anti-TNFα (from 1 to 16 μg/mL), and anti-IL-1β (from 1 to 16 μg/mL) for 24 h was measured by the MTS assay. Results are displayed as a percentage of the control group. The IC50 of CoCl_2_ was approximately 50 µM. The non-toxic concentrations of anti-TNFα and anti-IL-1β ranged from 1 to 4 μg/mL. (**E**) Concentration-dependent protective effect of anti-TNFα and anti-IL-1β (from 1 to 4 μg/mL) on CoCl_2_-injured BV-2 cell viability. (**F**) LDH release of BV-2 cells treated with 4 μg/mL anti-TNFα or anti-IL-1β for 24 h after exposure to 50 µM CoCl_2_ for 24 h. Data are expressed as the mean ± SD of four independent experiments. One-way ANOVA and post hoc Tukey’s corrections were used to calculate *p* values (* *p* < 0.01, ** *p* < 0.01, *** *p* < 0.001, and **** *p* < 0.0001).

The morphological features of CoCl_2_-injured BV-2 cells were observed by staining the cytoskeleton with fluorescein–phalloidin. Correspondingly, we noted distinct morphological changes in BV-2 microglia stressed with increasing concentrations of CoCl_2_, and the representative images are shown in Figure 1A. Untreated normal BV-2 cells (control) were uniform in size and displayed spherical, elliptical, or triangular shapes. CoCl_2_ stress resulted in ramified shapes of many BV-2 cells, indicating activation of the microglia. As the concentration of CoCl_2_ increased, the main bodies of the cells lost normal morphology, with increased and longer filopodia, extending to the surroundings.

BV-2 cells were exposed to different concentrations of CoCl_2_ (ranging from 10 to 120 μM) for 24 h. As shown in Figure 1B, the cell viability decreased in a concentration-dependent manner. Half of the maximum inhibitory concentration (IC50) of CoCl_2_ was designated as the optimal condition for simulating hypoxia in BV-2 cells. The results from the MTS assay demonstrated that the viability of BV-2 cells was close to 50% (51.1 ± 7.0%) when the concentration of CoCl_2_ was set at 50 µM, which has been shown to significantly increase HIF-1α in vitro in previous studies [20,21]; thus, this concentration was set for the following studies.

To determine the range of non-toxic concentrations of anti-TNFα and anti-IL-1β, BV-2 cells were exposed to different concentrations of anti-TNFα or anti-IL-1β (from 1 to 16 μg/mL). The MTS results showed that when the concentrations of anti-TNFα and anti-IL-1β were below 4 μg/mL, the cell viability was higher than 90% and was not significantly different from that of the control cells (Figure 1C,D). We excluded 8 μg/mL from the non-toxic concentration range of anti-TNFα and anti-IL-1β, given the decreased BV-2 cell viability (83.9 ± 3.1% and 80.9 ± 5.8%, respectively). This result revealed that anti-TNFα or anti-IL-1β at concentrations of 4 μg/mL and below are non-toxic for BV-2 cells.

Next, we evaluated the effects of anti-TNFα and anti-IL-1β on CoCl_2_-impaired cell viability. After exposure to 50 µM CoCl_2_, cells were treated with anti-TNFα and anti-IL-1β at different concentrations (1, 2, 4 μg/mL) for 24 h. The results of the MTS assay showed that anti-TNFα and anti-IL-1β improved cell viability in a concentration-dependent manner (shown in Figure 1E). With anti-TNFα or anti-IL-1β treatment at concentrations of 2 μg/mL and above, cell viability was significantly restored compared to that of the CoCl_2_ group. Both anti-TNFα and anti-IL-1β achieved the highest cell viability (90.6 ± 14.0% and 81.7 ± 10.4%, respectively) at a concentration of 4 μg/mL. This concentration is comparable to the antibody levels observed in patient serum when anti-cytokine therapy is administered systemically [22] or similar concentrations have been used in other in vitro experimental studies that have demonstrated its effectiveness [23]. For all subsequent experiments, BV-2 cells were treated with 4 μg/mL anti-TNFα or 4 μg/mL anti-IL-1β following exposure to CoCl_2_.

LDH, a soluble cytoplasmic enzyme, is rapidly released into the supernatant when the cytoplasmic membrane is disrupted, thereby serving as an indicator of cell injury. We investigated the effects of anti-TNFα and anti-IL-1β on CoCl_2_-induced hypoxic cytotoxicity. As expected, exposure to CoCl_2_ resulted in a dramatically increased release of LDH in the cell culture supernatant. This effect was substantially prevented by 4 μg/mL anti-TNFα or 4 μg/mL anti-IL-1β treatments (shown in Figure 1F). Together with improved cell viability, this result demonstrated that anti-TNFα and anti-IL-1β have remarkable protective effects on BV-2 microglia against CoCl_2_-induced hypoxic injury.

### 3.2. Anti-TNFα and Anti-IL-1β Treatment Alleviated CoCl_2_-Induced ROS Generation and Mitochondrial Impairment

**Figure 2:**
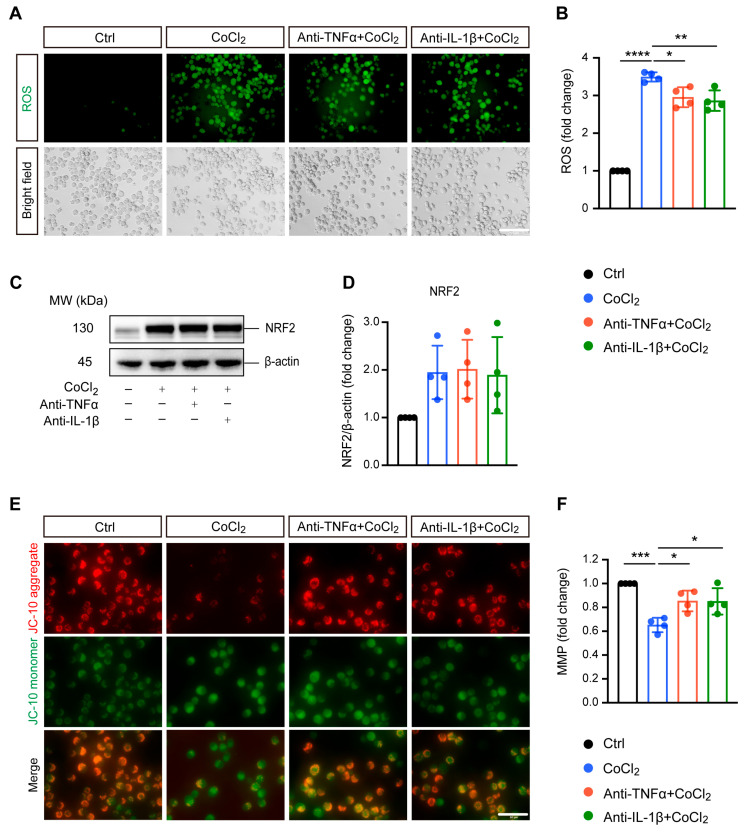
Anti-TNFα and anti-IL-1β alleviate CoCl_2_-induced oxidative damage in BV-2 cells. (**A**,**B**) Representative fluorescence images and quantification of intracellular reactive oxygen species (ROS) levels measured using the DCFH-DA probe under Ctrl, CoCl_2_, anti-TNFα + CoCl_2_, and anti-IL-1β + CoCl_2_ conditions (scale bar = 50 μm). (**C**,**D**) Representative Western blot images and quantification of NRF2 protein expression, with β-actin serving as the loading control. (**E**) Representative fluorescence images of mitochondrial aggregates (red) and monomers (green) using the JC-10 assay. The yellow color represents the overlap of red and green fluorescence (scale bar = 50 μm). (**F**) Statistical analysis showing that anti-TNFα and anti-IL-1β treatments restore mitochondrial membrane potential (MMP) levels in BV-2 cells, calculated based on the ratio of red to green fluorescence. Data are expressed as fold change relative to control cells, presented as mean ± SD from four independent experiments. Statistical significance was determined using one-way ANOVA with post hoc Tukey correction (* *p* < 0.05, ** *p* < 0.01, *** *p* < 0.001, **** *p* < 0.0001). Raw western blot image Figures S1 and S2.

Excessive ROS generation is implicated in hypoxia-induced cell damage, a common pathological process in neurodegenerative diseases [24]. In our study, DCFDA fluorescent staining revealed that exposing BV-2 cells to CoCl_2_ led to a significant increase in ROS levels compared to the control group. Treatment with anti-TNFα and anti-IL-1β slightly mitigated this elevated intracellular ROS production induced by CoCl_2_ (Figure 2A,B). We examined nuclear factor (erythroid-derived 2)-like 2 (NRF2), a classical antioxidant transcriptional factor. However, neither anti-TNFα nor anti-IL-1β significantly altered CoCl_2_-induced NRF2 expression (Figure 2C,D).

Mitochondria are considered the main site of ROS production, and increased intracellular ROS can directly lead to mitochondrial damage [25]. Therefore, we further evaluated MMP, which is a global indicator of mitochondrial function. In healthy cells, the JC-10 dye accumulates in the mitochondrial matrix, forming red fluorescent aggregates. When MMP collapses, JC-10 converts to its monomeric green fluorescent form in dysfunctional mitochondria. Thus, MMP was assessed by the ratio of red to green fluorescence. Our result showed that exposure to CoCl_2_ resulted in a sharp decline in MMP to 65.2% (*p* < 0.001) as compared to the control group. Anti-TNFα and anti-IL-1β treatment significantly alleviated the CoCl_2_-induced dissipation of red fluorescence in BV-2 cells, restoring MMP to 85.4% and 85.0%, respectively (*p* < 0.05) (Figure 2E,F). This suggests that anti-TNFα and anti-IL-1β treatments may exert their protective effects on BV-2 cells against CoCl_2_ primarily through mitochondrial stabilization rather than broad-spectrum antioxidation.

### 3.3. Anti-TNFα and Anti-IL-1β Reduced BV-2 Cell Apoptosis by Inhibiting ATF4 and p38 MAPK/Caspase-3 Pathways

**Figure 3:**
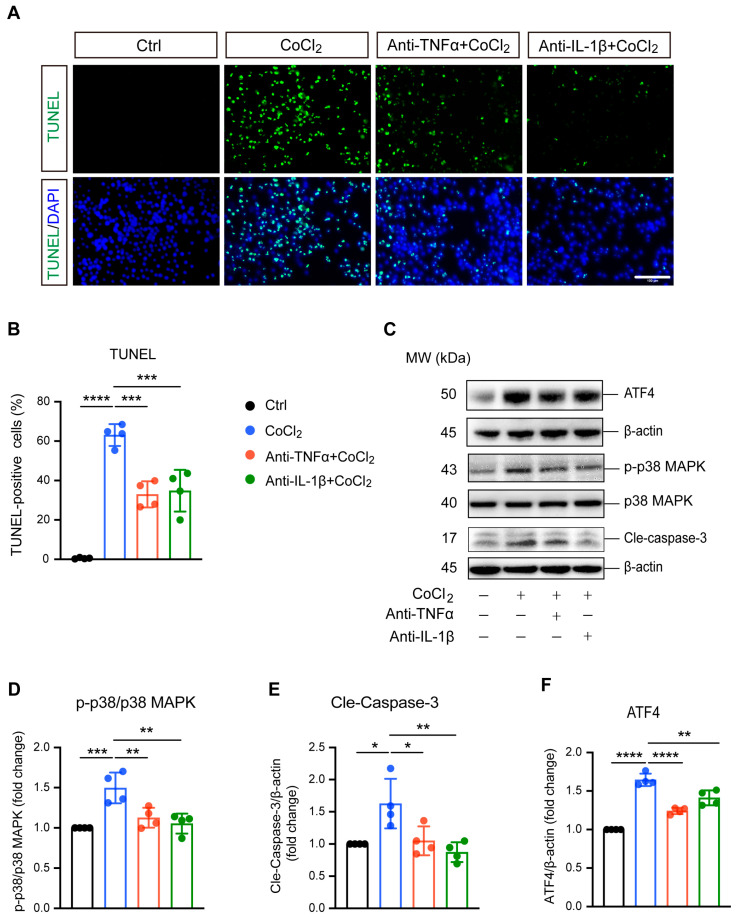
Anti-TNFα and anti-IL-1β inhibited CoCl_2_-induced apoptosis of BV-2 cells. (**A**) Representative fluorescence images of TUNEL staining (green) with DAPI nuclear counterstain (blue) across different treatment groups: Ctrl, CoCl_2_, anti-TNFα + CoCl_2_, and anti-IL-1β + CoCl_2_. Scale bar = 100 µm. (**B**) Quantification of apoptosis rates is shown as the percentages of TUNEL-positive cells relative to the total cell count. (**C**) Representative WB images displaying the expression levels of phosphorylated p38 MAPK (p-p38), total p38 MAPK, cleaved caspase-3 (Cle-Caspase3), and ATF4, with β-actin serving as the loading control. (**D**–**F**) Quantitative analyses of the ratios of p-p38 to total p38 MAPK, Cle-Caspase3, and ATF4 protein levels across the indicated groups. Data are expressed as the mean ± SD from four independent experiments. Statistical significance was determined using one-way ANOVA followed by post hoc Tukey’s test (* *p* < 0.05, ** *p* < 0.01, *** *p* < 0.001, and **** *p* < 0.0001). Raw western blot image Figures S3 and S4.

TUNEL staining and apoptosis-related proteins caspase-3, p38 MAPK, and phosphorylated p38 MAPK (p-p38 MAPK) were analyzed to evaluate apoptosis in BV-2 cells. As shown in Figure 3A, exposure to CoCl_2_ resulted in a significant increase in TUNEL-positive BV-2 cells, indicating elevated apoptosis. Quantitative analysis revealed that the CoCl_2_ group had a substantially higher percentage of apoptotic BV-2 cells compared to the control group (63.1% vs. 5.4%, *p* <0.0001) (Figure 3B). Treatment with anti-TNFα or anti-IL-1β lowered the apoptosis rate to 33.0% and 34.8%, respectively (*p* <0.001). Western blot analysis demonstrated that CoCl_2_ exposure significantly increased the ratio of p-p38 MAPK to p38 MAPK and elevated the levels of cleaved caspase-3 (Cle-Caspase3) (*p* <0.001 and *p* <0.05, respectively). These effects were effectively reversed by treatment with anti-TNFα and anti-IL-1β (Figure 3D,E). Additionally, we examined the expression of activating transcription factor 4 (ATF4), a key mediator in endoplasmic reticulum (ER) stress known to promote apoptosis and exacerbate neuronal damage [26]. Our results showed that CoCl_2_ led to a dramatic increase in ATF4 expression compared to the control group, whereas anti-TNFα and anti-IL-1β significantly downregulated CoCl_2_-induced ATF4 expression (Figure 3F). These results suggest that anti-TNFα and anti-IL-1β ameliorate CoCl_2_-induced BV-2 cell apoptosis by inhibiting ATF4 and p38 MAPK/caspase-3 pathways.

### 3.4. Anti-TNFα and Anti-IL-1β Suppress Hypoxia-Induced Microglial Reactivity by Inhibiting STAT1 and NF-κB/NLRP3 Pathways

**Figure 4:**
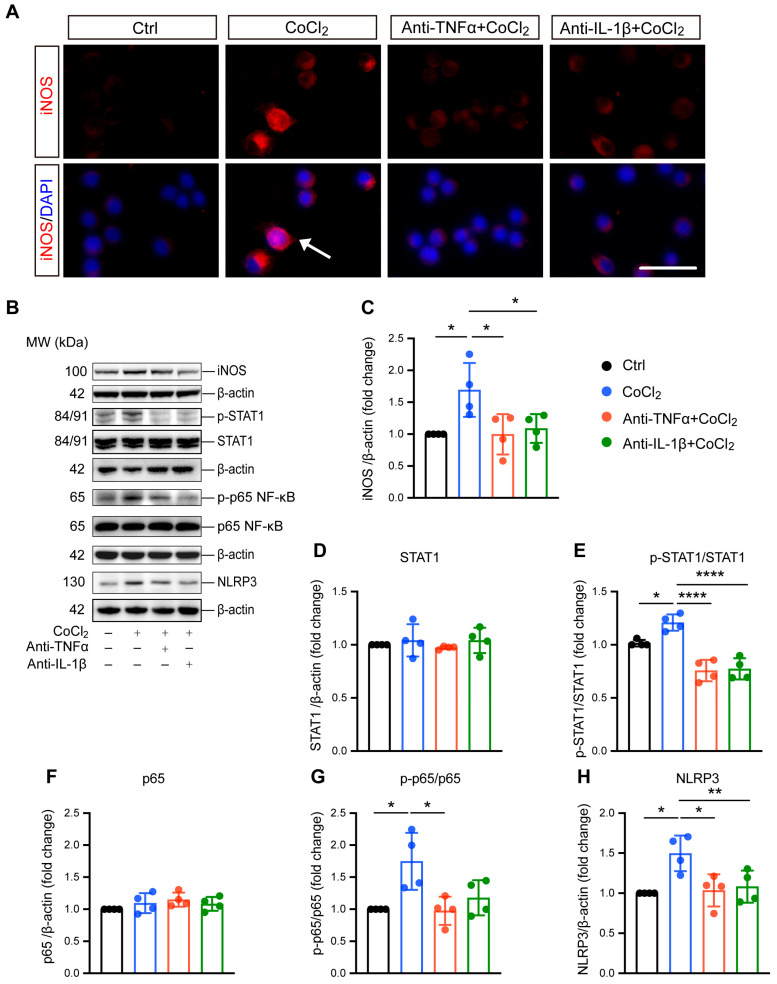
Anti-TNFα and anti-IL-1β inhibit CoCl_2_-induced microglial reactivity and suppress activation of inflammatory pathways in BV-2 cells. (**A**) Immunofluorescence images of BV-2 cells stained for iNOS (red) and counterstained with DAPI (blue). The white arrows indicate the cell exhibiting nuclear iNOS localization, suggesting nuclear translocation (scale bar = 25 μm). (**B**) Representative Western blot images displaying expression levels of iNOS, phosphorylated STAT1 (p-STAT1), total STAT1, phosphorylated NF-κB p65 (p-p65), total NF-κB p65, and NLRP3 across different treatment groups. (**C**) Quantitative analysis of iNOS expression levels normalized to β-actin, presented as fold change relative to the control group. (**D**–**H**) Quantitative analyses of the expression levels of p-STAT1, STAT1, p-p65, p65, and NLRP3, respectively. Data are normalized to corresponding total protein levels or β-actin and expressed as fold change relative to control. Data shown are the mean ± SD of four independent experiments. One-way ANOVA and post hoc Tukey’s corrections were used to calculate *p* values (* *p* < 0.05, ** *p* < 0.01, and **** *p* < 0.0001). Raw western blot Figures S5–S7.

Microglial reactivity is associated with neurotoxic effects and neuroinflammation [27]. Here, we investigated the effect of CoCl_2_-induced hypoxia on microglial reactivity, as well as the impacts of anti-TNFα and anti-IL-1β. Inducible nitric oxide synthase (iNOS) was used as the marker for this reactive state and has been implicated in microglial-associated neuroinflammation. Immunofluorescence staining and Western blot analysis consistently revealed a significant overexpression of iNOS (Figure 4A,B), accompanied by notable nuclear translocation (indicated by white arrow in Figure 4A). Treatment with anti-TNFα and anti-IL-1β effectively reduced iNOS expression (Figure 4A–C), indicating the suppression of microglial reactivity. To elucidate the mechanisms underlying hypoxia-induced microglial polarization, we examined the expression and phosphorylation of STAT1, a key transcription factor reported to be an essential mediator of pro-inflammatory microglial responses [28]. The results indicated CoCl_2_ exposure resulted in robust activation of STAT1, as evidenced by increased phosphorylation levels (Figure 4E). Importantly, both anti-TNFα and anti-IL-1β treatments prevented this activation, indicating their role in modulating STAT1 signaling pathways.

Microglial reactivity-induced neuroinflammation is closely related to NF-κB/NLRP3 signaling pathway activation. Therefore, we investigated the involvement of NF-κB in BV-2 cell response to hypoxia by evaluating the major NF-kB subunit p65. Our data showed that the ratio of phosphorylated p65 (p-p65) to p65 significantly increased in cells exposed to CoCl_2_, indicating the activation of NF-κB signaling. Both anti-TNFα and anti-IL-1β were able to abolish CoCl_2_-induced activation of NF-κB signaling, as indicated by reduced p-p65 expression (Figure 4B). Specifically, the p-p65/p65 ratio decreased from 1.75 ± 0.45 in the CoCl_2_ group to 0.97 ± 0.22 and 1.18 ± 0.27 in the anti-TNFα- and anti-IL-1β-treated groups, respectively (*p* < 0.05 for anti-TNFα; *p* = 0.06 for anti-IL-1β). NF-κB activation could induce the expression of NLRP3, a key factor in microglia-associated neuroinflammation [29]. We further examined the protein level of NLRP3, and the result showed that it was significantly upregulated in the CoCl_2_ group. However, this upregulation was reversed by anti-TNFα and anti-IL-1β treatments (Figure 4H). Collectively, our findings suggest that anti-TNFα and anti-IL-1β suppress pro-inflammatory microglial reactivity by inhibiting STAT1 activation and the NF-κB/NLRP3 signaling pathway.

### 3.5. Hierarchical Clustering Analysis of Differentially Expressed Proteins in BV-2 Cells

**Figure 5:**
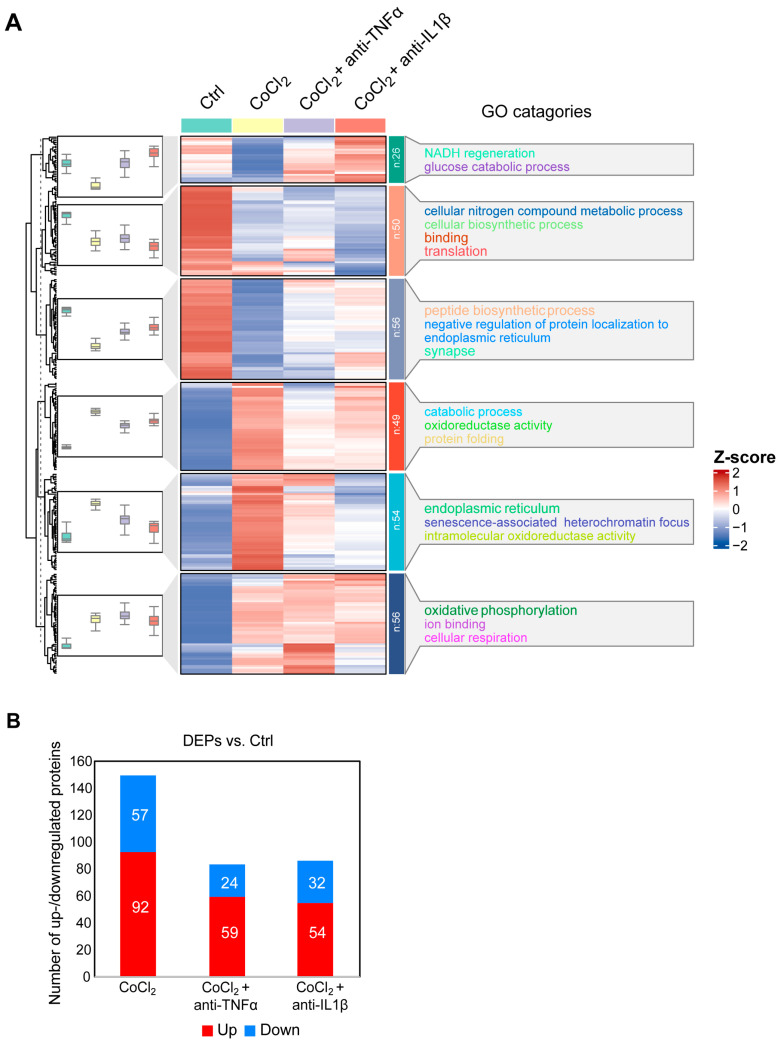
Overview of differentially expressed proteins from. (**A**) Hierarchical heatmap clustering of 292 significant proteins identified across four experimental groups: control, CoCl_2_, CoCl_2_ + anti-TNFα, and CoCl_2_ + anti-IL-1β. The heatmap displays relative z-scores, with red indicating upregulation and blue indicating downregulation. On the right side of the heatmap, there are the word cloud annotations that summarize the main functions in every GO cluster. (**B**) Graph presenting the number of significantly up- (red) and downregulated (blue) proteins in experimental groups compared with those of control (*p* < 0.05 and |log2FC| > 1).

To gain a comprehensive understanding of the molecular alterations in BV-2 microglial cells under hypoxic conditions and the effects of anti-TNFα and anti-IL-1β treatments, we conducted a label-free quantitative mass spectrometry (MS) analysis. This analysis compared four groups: control, CoCl_2_, CoCl_2_ + anti-TNFα, and CoCl_2_ + anti-IL-1β. A total of 292 proteins exhibited differential expression across these groups (*p* < 0.05). Hierarchical clustering of these proteins was visualized using a heatmap (Figure 5A), which revealed distinct clustering patterns among the groups. To identify proteins with significant changes in expression, we applied a fold change (FC) threshold of >2 and a *p*-value <0.05. Compared to the control group, 149 differentially expressed proteins (DEPs) were identified in BV-2 cells exposed to CoCl_2_; of these, 92 proteins were upregulated, and 57 were downregulated. Notably, the number of DEPs dramatically declined in the CoCl_2_ + anti-TNFα and CoCl_2_ + anti-IL-1β groups compared to the CoCl_2_ group (Figure 5B). In the CoCl_2_ + anti-TNFα group, 59 DEPs were upregulated and 24 downregulated, while in the CoCl_2_ + anti-IL-1β group, 54 DEPs were upregulated and 32 downregulated compared to the control group.

### 3.6. Gene Ontology Classification and Key Proteins Involved in Hypoxia-Damaged BV-2 Microglia

**Figure 6:**
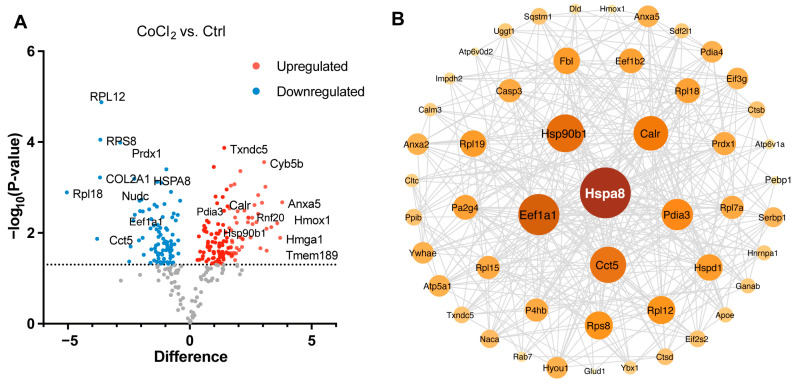

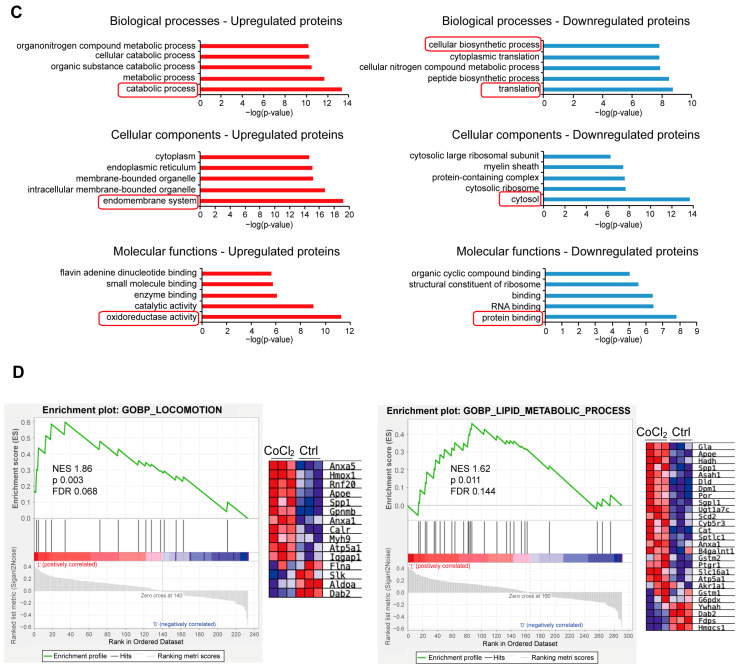
Overview of differentially expressed proteins from profiling of differentially expressed proteins between the CoCl_2_ group and the control group (*n*  =  3/group). (**A**) Volcano plot showing up- (red) and downregulated (blue) proteins in the CoCl_2_-induced hypoxia group compared with those of the control. The *X*-axis shows the log2 FC. The *Y*-axis shows the negative logarithm of the *p*-value. (**B**) Protein–protein interaction (PPI) network by Cytoscape: node size and color represent the degree. The node size is proportional to its closeness centrality. (**C**) Gene ontology (GO) distribution based on biological processes, cellular components, and molecular functions of up- (red) and downregulated (blue) expressed proteins in BV-2 cells damaged by CoCl_2_, compared with those of the control. The main GO terms of interest are highlighted by red rectangles. The false discovery rate (FDR) < 0.05. (**D**) Gene set enrichment analysis (GSEA) demonstrates that the locomotion and lipid metabolic process are enriched in the CoCl_2_-damaged group. The heatmaps show the core enrichment genes. *p* <  0.05, FDR < 0.25.

We performed a detailed examination of the significantly changed proteins in the CoCl_2_ group, compared to the control (*p* < 0.05). Volcano plots showed the up-/downregulated proteins in the CoCl_2_ group (Figure 6A), among which HSPA8, eEF1A1, CCT5, and the ribosomal proteins, including RPS8, RPL12, and RPL 18, were significantly downregulated. HSP90B1, CALR, and PDIA3 were upregulated. Notably, protein–protein interaction (PPI) network using Cytoscape (version 3.10.2) analyses revealed that HSPA8 was the central node, and eEF1A1, HSP90B1, CALR, CCT5, and PDIA3 were important nodes (Figure 6B). To understand the function, location, and biological pathway of the DEPs, we conducted Gene Ontology (GO) analysis based on the biological process, cellular component, and molecular function using the Panther Classification System (version 15.0). Figure 6C showed that the DEPs between the CoCl_2_ group and the control group were classified as the top five GO terms based on –log10 (*p* value). The upregulated proteins of the CoCl_2_-treated BV-2 cells were mainly involved in the catabolic process, endomembrane system, and oxidoreductase activity. The downregulated proteins were related to cellular biosynthetic process, translation, cytosol, and protein binding. In addition, gene set enrichment analysis (GSEA) showed that the locomotion and lipid metabolic process pathways were enriched in the CoCl_2_-stressed group (Figure 6D). Proteins upregulated in the CoCl_2_ group included ANXA5 and APOE, suggesting their potential involvement in the stress response to hypoxia.

### 3.7. Network Analysis of Recovery Process Mediators

**Figure 7:**
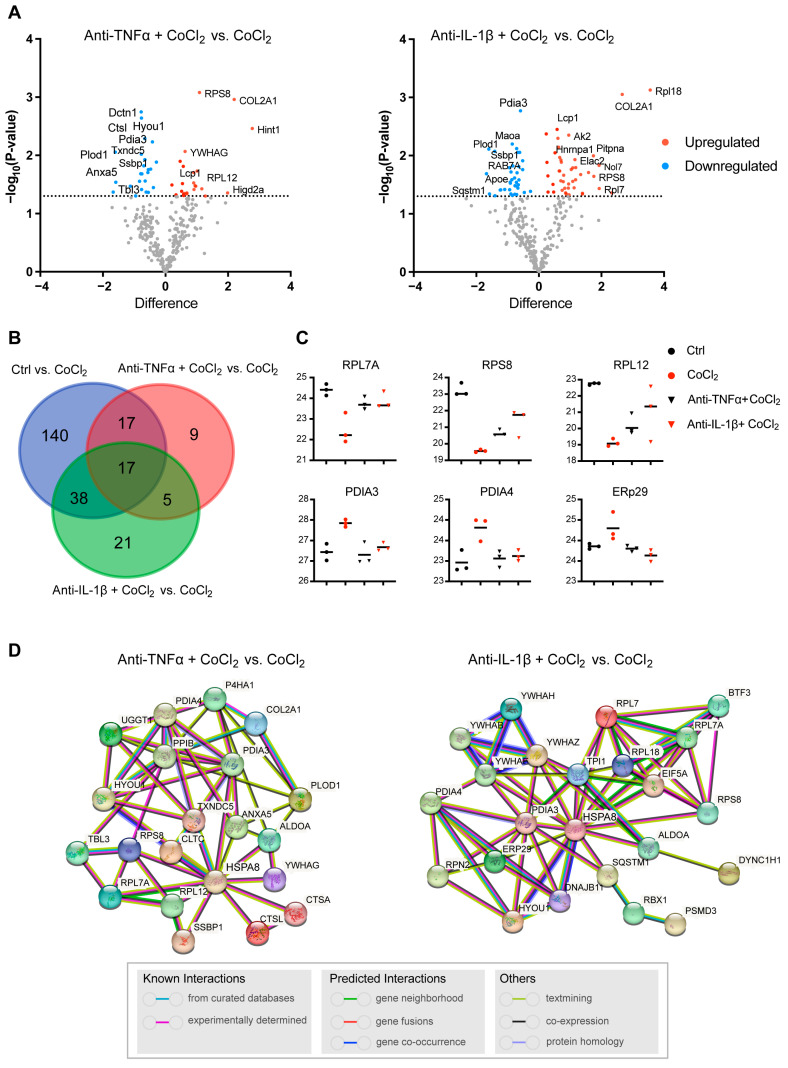
Profiling of DEPs of BV-2 cells in CoCl_2_ + anti-TNFα group and CoCl_2_ + anti-IL-1β group, compared with those of CoCl_2_ group (*n*  =  3/group). (**A**) Volcano plots of differential protein expression in CoCl_2_ + anti-TNFα and CoCl_2_ + anti-IL-1β vs. CoCl_2_-treated cells. The *X*-axis shows the log2 FC. Proteins reduced are negative and upregulated genes positive. The *Y*-axis shows the negative logarithm of the *p*-value. Grey dots represent proteins that were not significantly differentially expressed. (**B**) Venn diagram of significantly DEPs compared to CoCl_2_ group. The criterion for protein inclusion was a *p* < 0.05. (**C**) Scatter plots showing the expression levels of ribosomal and ER-related proteins. (**D**) Signaling pathways altered by anti-TNFα and anti-IL-1β treatment were selected based on the lowest false discovery rate and analyzed by STRING protein interaction network.

To further illustrate the recovery mechanism, we examined the DEPs in the CoCl_2_ + anti-TNFα group and the CoCl_2_ + anti-IL-1β group, compared with the CoCl_2_ group based on a *p* < 0.05. Volcano plots showed the up-/downregulated proteins in the CoCl_2_ + anti-TNFα group and CoCl_2_ + anti-IL-1β group, respectively (Figure 7A). Notably, ANXA5 and APOE were among the proteins downregulated by anti-TNFα and anti-IL-1β, respectively. A Venn diagram identified 17 co-expressed DEPs among the control, CoCl_2_ + anti-TNFα, and CoCl_2_ + anti-IL-1β, 9 unique DEPs in CoCl2 + anti-TNFα, and 21 unique DEPs in CoCl_2_ + anti-IL-1β (Figure 7B). A total of 48 proteins changed their expression after anti-TNFα treatment; 81 proteins changed their expression after anti-IL-1β treatment (Figure 7B). Anti-TNFα and anti-IL-1β treatment restored the expression of ribosomal proteins such as RPL7A, RPS8, RPL12, and RPL18, and decreased CoCl_2_-induced ER-related proteins, such as PDIA3, PDIA4, and ERp29 (Figure 7C). Protein–protein interaction (PPI) network analyses of related proteins were constructed using the STRING database (Figure 7D). Interestingly, HSPA8 is again shown as the center node, in the networks of both anti-TNFα-induced DEPs and anti-IL-1β-induced DEPs. Our results highlighted the proteins reported to be interacting with HSPA8, PDIA3, RPS8, ANXA5, HYOU1, PPIB, and TXNDC5 (Figure 7D).

### 3.8. Anti-TNFα, but Not Anti-IL-1β, Inhibited Hypoxia-Promoted Microglia Migration

**Figure 8:**
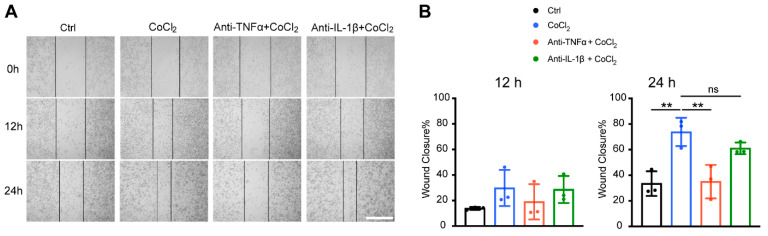
Anti-TNFα inhibited CoCl_2_-induced BV-2 cell migration. (**A**) Representative images of scratch wound healing assay at 0 h, 12 h, and 24 h from the indicated groups (scale bar = 400 μm). (**B**) Wound closure rates of different groups at 12 h and 24 h. Data shown are the mean ± SD of three independent experiments; for each experiment, at least seven observations per group were recorded and then averaged. One-way ANOVA and post hoc Tukey’s corrections were used to calculate *p* values (** *p* < 0.01, ns = not statistically significant).

To further validate microglial motility, we assessed the microglia migration capacity under CoCl_2_ exposure with a scratch wound healing assay (wound closure % = (A0 − At)/A0%). The result showed that within 12 h of treatment, there was no significant difference in wound closure among the four groups (Ctrl, CoCl_2_, CoCl_2_ + anti-TNFα, and CoCl_2_ + anti-IL-1β). By 24 h, the wound closure rate in the CoCl_2_ group (73.8 ± 11.1%) was significantly higher than that in the control group (33.5 ± 9.6%) (*p* = 0.005) (Figure 8B), indicating that microglia migration was accelerated under the hypoxic condition, which was consistent with the proteomic analysis. Notably, the wound closure rate was significantly decreased by the anti-TNFα treatment (*p* = 0.007), but not by the anti-IL-1β treatment (*p* = 0.45) (Figure 8B).

## 4. Discussion

Hypoxia characterizes many chronic neurodegenerative diseases, including glaucoma, diabetic retinopathy, and other forms of retinal neuron loss. However, the pathological mechanisms of hypoxia-related retina diseases are highly complicated and remain to be elucidated. In this study, we showed that CoCl_2_-induced hypoxia stress led to microglia oxidative death and reactivity. This is the first study demonstrating that the monoclonal antibodies, anti-TNFα and anti-IL-1β, confer remarkable protection on BV-2 microglia against hypoxia-induced injury. Anti-TNFα and anti-IL-1β reduced oxidative stress with mitigated ROS generation and mitochondrial function impairment and reversed hypoxia-stimulated microglial reactivity by suppressing the activation of the STAT1, NF-κB, and NLRP3 inflammatory pathways. In addition, our MS-based proteomic analysis unraveled the underlying biochemical mechanisms in an unbiased way. HSPA8 was revealed as a central node in the network of proteins involved in pathological processes caused by CoCl_2_, and also acts as one of the major mediators of the recovery processes induced by anti-TNFα and anti-IL-1β. Lastly, we found that microglia migration was accelerated upon hypoxia, which can be reversed by treatment with anti-TNFα but not anti-IL-1β. These findings provide new insights into the antioxidant and anti-inflammatory mechanisms of anti-TNFα and anti-IL-1β on microglia, highlighting their potential as protective agents against neurodegenerative diseases.

The neuroprotective effects of anti-TNFα and anti-IL-1β have attracted increasing interest. Recent studies utilizing animal models have shown the capacities of anti-TNFα and anti-IL-1β in attenuating neurological deficits in Alzheimer’s disease, Parkinson’s disease, and ischemic stroke [30,31,32]. Nevertheless, these in vivo animal studies failed to specify the protective effects of anti-TNFα and anti-IL-1β in distinct cell populations. We applied an in vitro cell model to investigate the direct protective effects of anti-TNFα and anti-IL-1β specifically on microglia. A chemical hypoxic microglia model was established in BV-2 cells using CoCl_2_, a well-known hypoxia-mimetic agent. In our study, anti-TNFα and anti-IL-1β protected BV-2 cell viability against hypoxia in a dose-dependent manner. The optimal concentration (4 μg/mL) of anti-TNFα and anti-IL-1β remarkably restored cell viability (Figure 1E). Hypoxia, due to the lack of oxygen (O_2_) as the electron recipient, leads to mitochondrial ROS accumulation, and mitochondria themselves are also a major target of oxidative damage [25]. Mitochondrial dysfunction is an underlying feature of the injury cascade following hypoxia [33]. Previous evidence has indicated that both pharmacological modulation of mitochondrial function and direct transplantation of functional mitochondria display therapeutic potential in the treatment of neurodegenerative diseases [34,35]. In our study, the anti-TNFα and anti-IL-1β treatment alleviated ROS generation partially, while restoring MMP remarkably. Specifically, the protective effects mediated by anti-TNFα and anti-IL-1β were related to the regulation of ATF4, rather than the classical antioxidant transcriptional factor NRF2 (Figure 2). The role of ATF4 differs in different cells under oxidative stress. In neurons and glial cells, ATF4 is a key prodeath transcriptional activator [26] and overexpression of ATF4 is associated with retina and brain degeneration in murine models [36,37]. Both pharmacological blocking and genetic silencing of ATF4 have been shown to promote neuron survival [26,38]. In our study, anti-TNFα and anti-IL-1β significantly decreased hypoxia-induced ATF4 expression, suggesting that anti-TNFα and anti-IL-1β enhance the resistance of BV-2 microglia to oxidative stress by inhibiting ATF4.

Anti-TNFα and anti-IL-1β treatment prevented CoCl_2_-induced BV-2 cell death, as evidenced by reduced numbers of TUNEL+ cells and decreased protein expression of the pro-apoptotic regulators, phospho-p38 MAPK and cleaved caspase-3. Activation of p38 MAPK is considered to be involved in the pathogenesis of retinal neurodegenerative diseases [39]. This is supported by our results showing that CoCl_2_-induced BV-2 cell death was accompanied by a significant increase in the phosphorylation activation of p38 MAPK (Figure 3D). Once activated, p38 MAPK on the one hand aggravates neuroinflammation by activating NF-κB and on the other hand triggers cell death as a key mediator of apoptosis [40]. Phosphorylated p38 MAPK initiates activation of Caspase 3, the executor of apoptosis, via the extrinsic and intrinsic pathway, leading to apoptosis and cell death [41]. In this study, CoCl_2_-induced p38 MAPK phosphorylation and cleaved caspase-3 expression were markedly abrogated by anti-TNFα and anti-IL-1β, respectively. This can be partially explained by the possible neutralization of the neurotoxic effects of TNFα and IL-1β, which directly activate apoptotic and necroptotic pathways [42,43] and mediate the abnormalities of glutamate transmission [44,45]. Overall, our results suggest that anti-TNFα and anti-IL-1β represent a promising therapeutic strategy to prevent cell death under hypoxia.

Another important result of our study is that microglia can exhibit reactive states in response to CoCl_2_-simulating hypoxia. As the first defense, microglia are highly sensitive and modify their morphology in response to various pathological stimuli. Here, we recorded distinctive morphological changes in BV-2 cells under hypoxia, from a compact round shape to a ramified shape, with branches elongated and extended (Figure 1A). Recent studies reveal that highly ramified microglia are dynamic and their processes are continuously moving [46,47]. The CoCl_2_-induced morphological characteristics in BV-2 cells are in accordance with those observed in LPS-stimulated microglia, suggesting that hypoxia induces a reactive response in these cells. The use of a simplified M1/M2 dichotomy for describing microglial activation is increasingly being questioned, due to the recognition that microglial states are highly dynamic and context-dependent. The consensus in the field is that the terminology surrounding microglial phenotypes requires refinement, with a focus on microglial states rather than fixed phenotypes [48]. Classically pro-inflammatory microglia reactivity, previously referred to as the M1 phenotype, has been extensively studied due to its critical role in aggravating neuroinflammation and neurotoxicity. The majority of these studies have focused on inflammatory stimuli. However, hypoxia-induced microglia reactivity is rarely investigated. By means of an in vitro model mimicking hypoxic injury, we observed significantly increased levels of iNOS, a marker traditionally associated with a pro-inflammatory state of microglia, in CoCl_2_-stressed BV-2 cells. Our study confirmed that hypoxia is sufficient to drive microglial reactivity to a pro-inflammatory state. To further elucidate the mechanism, we examined STAT1, which has been proposed as an essential transcription factor that regulates hypoxia-induced microglial reactivity [49]. Recent studies utilizing in vivo and in vitro hypoxia models [49,50] reported that activation of the STAT1-related pathway leads to a pro-inflammatory microglial reactivity. Furthermore, silencing STAT1 or inhibiting its activity can counteract this hypoxia-induced reactivity [49,51]. In agreement with these studies, our study revealed that CoCl_2_-induced hypoxia led to an increased STAT1 phosphorylation level, which was reversed by anti-TNF-α and anti-IL-1β thereby inhibiting hypoxia-induced microglial reactivity.

CoCl_2_ induced an increase in the phosphorylation of NF-κB (Figure 4G), a pivotal mediator of microglial-mediated neuroinflammation following hypoxic injury [52]. NF-κB activation leads to the expression of pro-inflammatory cytokines such as TNF-α and IL-1β [53], and also contributes to the production of ROS, creating a vicious cycle where oxidative stress induces inflammation and vice versa [54]. Upon binding to their respective receptors (TNFR and IL-1R), both TNF-α and IL-1β can instigate an intracellular signal transduction pathway activating NF-κB [55,56], triggering various transcriptions of pro-inflammatory genes [57]. Previous studies have demonstrated that inhibiting TNF-α and IL-1β binding to their receptors, either through pharmacological blockade or gene silencing, effectively suppresses NF-κB pathway activation [58,59]. In agreement with these studies, our results showed that anti-TNF-α and anti-IL-1β treatment significantly inhibited NF-κB p65 activation under hypoxia. The suppression led to reduced expression of pro-inflammatory mediators and cytokines, attenuating the excessive inflammatory response triggered by microglia [60]. Additionally, NF-κB inhibition can decrease NADPH oxidase subunit gp91phox (No x_2_) expression, reducing oxidative stress [61], which may have contributed to the observed ROS reduction and MMP preservation following anti-TNF-α and anti-IL-1β treatment in our BV-2 model. Furthermore, we observed downregulation of NLRP3, a key downstream molecule of NF-κB. Upregulated NLRP3 is involved in retina neurodegeneration owing to its contribution to facilitating pyroptosis and intensifying neuroinflammation [62,63]. In return, NLRP3 inflammasome is also a crucial inducer of pro-inflammatory microglial state transition [62]. Therefore, inhibition of the NF-κB/NLRP3 signaling pathway is another mechanism by which anti-TNF-α and anti-IL-1β reverse microglial reactivity. Taken together, anti-TNF-α and anti-IL-1β mediate anti-neuroinflammatory effects by inhibiting the positive feedback loop between microglial reactivity and inflammatory pathway activation.

Proteomic analysis assessed the integrated impact of CoCl_2_ on BV-2 cells, suggesting that hypoxia prompts microglia senescence. This is evident from the results showing that downregulated proteins in the CoCl_2_ group are associated with the GO terms translation and peptide biosynthetic process, while upregulated proteins are involved in the catabolic process, among others (Figure 6C). Aging is accompanied by a decline in cellular proteostasis caused by the functional degradation of ribosomes [64]. We found that among the key downregulated candidate proteins in CoCl_2_-treated cells, numerous ribosomal RPS and RPL family proteins are ribosome-related proteins with pivotal functions in protein synthesis and metabolic processes. Impaired protein synthesis is one of the earliest abnormalities in neurodegenerative diseases [65] and decreased ribosomal RNA was found in the brains of Alzheimer’s disease (AD) patients [66]. In this study, ribosome-related proteins such as RPS8, RPL12, and RPL7A were found in the DEP lists of upregulated proteins in the anti-TNF-α- and anti-IL-1β-treated groups. Regarding proteins upregulated by CoCl_2_, we found a series of proteins related to endoplasmic reticulum stress (ERS). Similar findings were reported by Wang et al., who observed that hypoxia increased the ER stress-triggered unfolded protein response accompanied by elevated ER-resident chaperone levels [67]. Our proteomic analysis is consistent with these results. Additionally, the ERS-related proteins were downregulated following anti-TNF-α and anti-IL-1β treatment, including those implicated in neurological disorders such as HYOU1 [68], PDIA4 [69], and PDIA3 [70]. Other proteins downregulated in the anti-TNFα + CoCl_2_ group include new signature proteins of AD such as ANXA5 [71] and PLOD1 [72]. Interestingly, APOE was specifically downregulated in the anti-IL-1β+ CoCl_2_ group. It has been reported that APOE is involved in dysfunctional microglia in neurodegenerative diseases [73]. Our data therefore suggest that anti-TNF-α and anti-IL-1β treatment leads to beneficial effects against senescence, such as alleviating ribosome dysfunction and ER stress, as demonstrated by reversing hypoxia-induced biological changes at the molecular level. To further reveal the underlying mechanism, we performed the PPI networks analysis. It is worth noting that HSPA8 was identified as a central mediator, not only in the networks of hypoxic injury (Figure 6B), but also in recovery processes (Figure 7D). HSPA8 is a constitutively expressed protein that ensures cellular integrity against stressors [74]. Recent research by Wu et al. revealed distinct properties of HSPA8 in acting as an amyloidase, dismantling functional amyloids and inhibiting necroptosis signaling [75]. Taken together, this suggests that HSPA8 is a highly attractive new target for future research on neurodegenerative diseases.

The migration of activated microglia to the ongoing retinal lesion is recognized as a hallmark of pathogenesis in retinal neurodegenerative diseases [76]. In this study, proteomic analysis showed that the locomotion pathway was enriched in the CoCl_2_-stressed group (Figure 6D). By a scratch wound assay, we further verified that microglia migration was accelerated upon CoCl_2_-induced hypoxia. Similar results were also reported by Wang et al., who observed that chronic intermittent hypoxia promoted microglial migration in vivo and in vitro [77]. In fact, activated microglia migrate to inflammatory sites via an extension of their processes, amplifying neuroinflammation and exerting neurotoxic effects [78]. The accelerated migration of BV-2 cells was in accordance with the transition to ramified and elongated morphology in response to CoCl_2_-induced hypoxia (Figure 1A). Interestingly, we found that hypoxia-enhanced microglial migration can be significantly reversed by treatment with anti-TNFα but not anti-IL-1β. The differential effects of anti-TNFα and anti-IL-1β on microglial migration, despite their shared ability to inhibit inflammation and apoptosis, reflect the distinct roles of these cytokines in cellular behavior. According to previous studies, TNFα may directly promote migration by acting as a chemoattractant [79,80], inducing chemokines such as CCL2 and CXCL12 [81,82], activating matrix metalloproteinases (MMP-2 and MMP-9) to remodel the extracellular matrix [83], and regulating cytoskeletal dynamics via PI3K/Akt and Rho GTPases [84]. In our study, we also found that anti-TNFα downregulates the expression of ANXA5 (Figure 7A), a protein found to be enriched in the gene set related to locomotion in the CoCl_2_ group, according to our GSEA analysis (Figure 6D). However, the role of ANXA5 in migration remains uncertain, and further studies are needed to elucidate its potential impact on this process.

Some limitations of this study should be considered. Our in vitro approach does not reproduce the retina environment, and other pathogenic factors influencing microglial viability and function are absent. Beyond the scope of the methodologies published in this study, applications of alternative neurodegeneration induction methods in ex vivo or in vivo models can be used for further investigations. The therapeutic efficacy of anti-TNF-α and anti-IL-1β in vivo will depend upon optimized doses that block excessive release of TNF-α and IL-1β without significantly affecting their physiological functions such as local immunity. Introducing anti-TNF-α and anti-IL-1β therapy after substantial neurodegeneration has already happened may not be able to achieve significant neuroprotection, considering the involvement of other cytotoxic substances and a highly amplified apoptosis cascade at this stage. In addition, the microglial proteomic changes in this study cannot directly resolve the pattern of retinal proteomic changes. To address this, future studies may consider combining bulk and single-cell proteomes to clarify the overall effects on the retina and the specific effects on individual retinal cells. Furthermore, engagement of Fc receptors may also affect the efficacy of anti-cytokine antibody therapy. Therefore, IgG isotype controls should be included in future studies to better validate the specificity effects.

## 5. Conclusions

In conclusion, this study provides a comprehensive analysis of the mechanisms and proteomic changes in microglia exposed to hypoxia-induced injury. Hypoxic conditions triggered both self-inflicted damage and inflammatory activation in microglia. We highlight a novel therapeutic strategy involving monoclonal antibodies, anti-TNF-α and anti-IL-1β, which restore microglial homeostasis by reversing oxidative, apoptosis, and inflammatory cascades. This protective effect is driven by the modulation of key signaling pathways, including ATF4, p38 MAPK, STAT1, and NF-κB/NLRP3. Additionally, proteomic analysis revealed that anti-TNF-α and anti-IL-1β treatments counteract hypoxia-induced senescence by modulating ribosomal and ER-associated proteins. Notably, HSPA8 was identified as the central node in the protein–protein interaction network, offering promising targets for future research. Overall, our findings enhance the understanding of hypoxia’s effects on microglial proteomic profiles and underscore the potential of cytokine-targeting monoclonal antibodies as a therapeutic strategy for neurodegenerative diseases.

## Data Availability

The raw data supporting the conclusions of this article will be made available by the authors on request.

## Supplementary Materials

The following supporting information can be downloaded at: https://www.mdpi.com/article/10.3390/antiox14030363/s1, Figures S1–S7: Raw western blot images.

## Abbreviations

The following abbreviations are used in this manuscript:

AD	Alzheimer’s disease
ANXA5	Annexin A5
APOE	Apolipoprotein E
ATF4	Activating transcription factor 4
CoCl_2_	Cobalt chloride
Ctrl	Control
DEP	Differentially expressed protein
ER	Endoplasmic reticulum
HSPA8	Heat shock protein family A (Hsp70) member 8
HYOU1	Hypoxia upregulated 1
IL-1β	Interleukin-1 beta
IL-1R	Interleukin-1 receptor
iNOS	Inducible nitric oxide synthase
LPS	Lipopolysaccharide
MAPK	Mitogen-activated protein kinase
MMP	Mitochondrial membrane potential
MS	Mass spectrometry
NF-κB	Nuclear factor kappa B
NLRP3	NLR family pyrin domain-containing 3
NRF2	Nuclear factor erythroid 2-related factor 2
PDIA3	Protein disulfide-isomerase A3
PDIA4	Protein disulfide-isomerase A4
PLOD1	Procollagen-lysine,2-oxoglutarate 5-dioxygenase 1
PPI	Protein–protein interaction
PPIB	Peptidylprolyl isomerase B
ROS	Reactive oxygen species
RPL7A	Ribosomal protein L7a
RPL12	Ribosomal protein L12
RPL18	Ribosomal protein L18
RPS8	Ribosomal protein S8
STAT1	Signal transducer and activator of transcription 1
TNF-α	Tumor necrosis factor alpha
TNFR	Tumor necrosis factor receptor
TUNEL	Terminal deoxynucleotidyl transferase dUTP nick end labeling
TXNDC5	Thioredoxin domain-containing protein 5

## Appendix A

**Table A1:** Antibodies used in this study.

Antibody	Host	Working Dilution	Supplier	RRID
TNFα	Armenian hamster	1:12.5	Thermo Fisher Scientific, Waltham, MA, USA.	AB_468491
IL-1β	Armenian hamster	1:12.5	Thermo Fisher Scientific, Waltham, MA, USA.	AB_468396
NLRP3	Rabbit	1:1000	Thermo Fisher Scientific, Waltham, MA, USA.	AB_2224377
β-actin	Rabbit	1:2000	Thermo Fisher Scientific, Waltham, MA, USA.	AB_10855480
NF-κB p65	Mouse	1:1000	Cell Signaling Technology, Danvers, MA, USA.	AB_10828935
phospho-NF-κB	Rabbit	1:1000	Cell Signaling Technology, Danvers, MA, USA.	AB_331284
STAT1	Rabbit	1:1000	Cell Signaling Technology, Danvers, MA, USA.	AB_2198300
phospho-STAT1	Rabbit	1:1000	Cell Signaling Technology, Danvers, MA, USA.	AB_10950970
iNOS	Rabbit	1:1000	Proteintech, Rosemont, IL, USA.	AB_2782960
p38 MAPK	Rabbit	1:1000	Abclonal, Woburn, MA, USA.	AB_2863345
phospho-p38 MAPK	Rabbit	1:1000	Abclonal, Woburn, MA, USA.	AB_2864024
ATF4	Rabbit	1:1000	GeneTex, Irvine, CA, USA.	AB_1240487
NRF2	Rabbit	1:1000	Proteintech, Rosemont, IL, USA.	AB_2782956
anti-rabbit (HRP) secondary antibody	Goat	1:8000	Abcam, Cambridge, UK.	AB_955447
iNOS	Mouse	1:200	Santa Cruz, Dallas, TX, USA.	AB_627810
anti-mouse secondary antibody (Alexa Fluor^®^ 488)	Goat	1:400	Thermo Fisher Scientific, Waltham, MA, USA.	AB_2895153
