# Supplementary material for: Anti-TNFα and Anti-IL-1β Monoclonal Antibodies Preserve BV-2 Microglial Homeostasis Under Hypoxia by Mitigating Inflammatory Reactivity and ATF4/MAPK-Mediated Apoptosis
Source: Antioxidants (Basel). 2025 Mar 19;14(3):363. doi: 10.3390/antiox14030363 (PMC11939723; doi:10.3390/antiox14030363)
Supplement: Supplementary file 1 [file antioxidants-14-00363-s001.zip › antioxidants-3502466-supplementary.pdf]

Original Western Blot Images

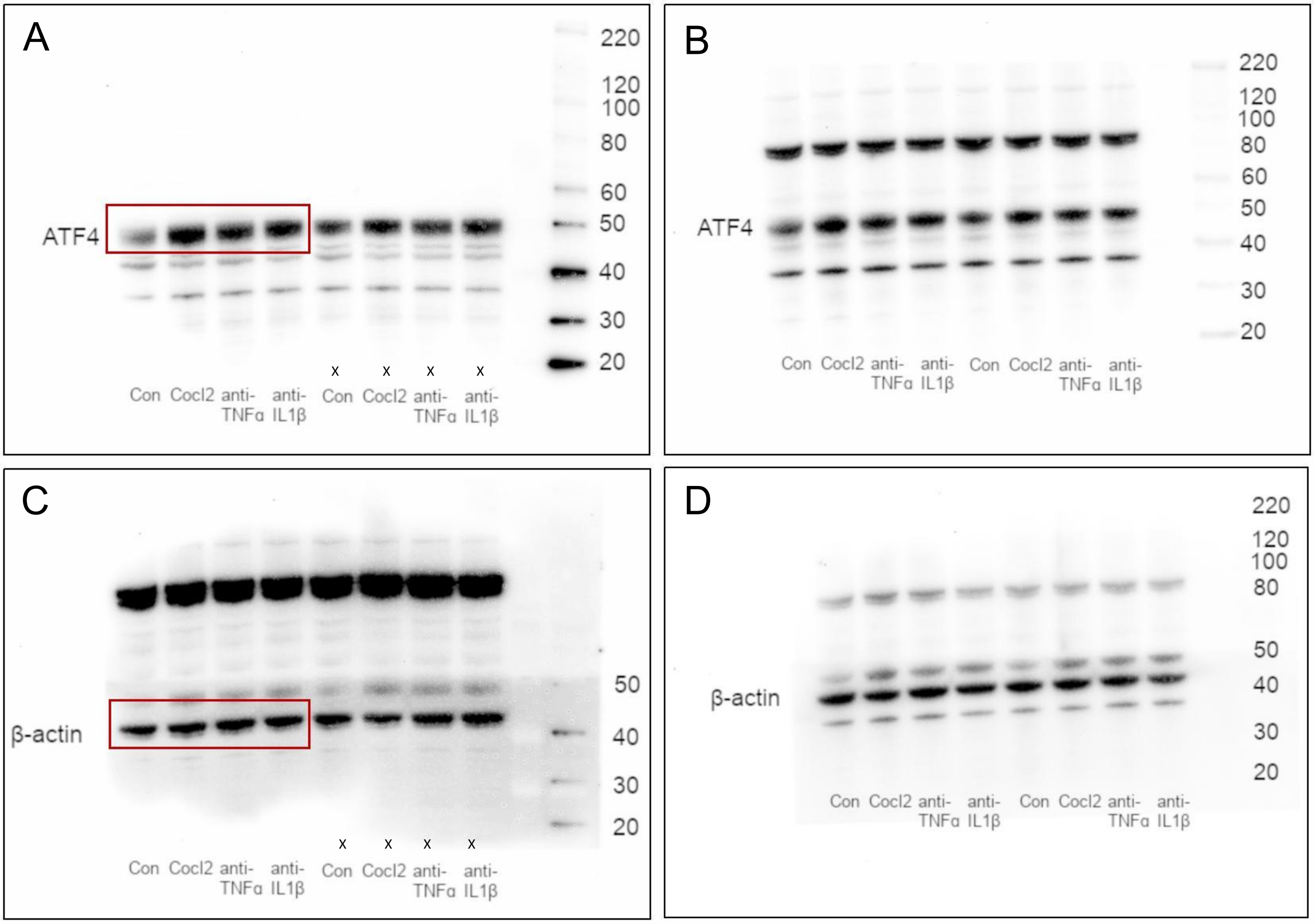

Figure S1. The entire uncropped images of the western blot for the protein ATF4 and the corresponding β-actin are shown in A – D, including four independent biological replicates. The bands marked by the red box in A and C are represented in the figure 2 of the manuscript.

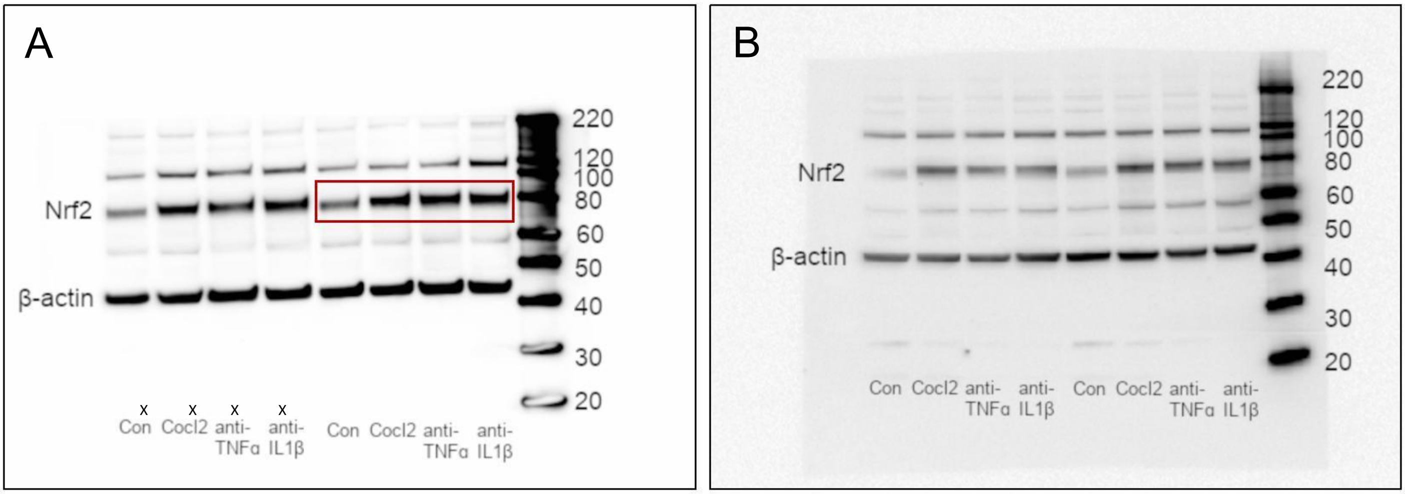

Figure S2. The entire uncropped images of the western blot for the protein Nrf2 and β-actin are shown in A – B, including four independent biological replicates. The bands marked by the red box in A are represented in the figure 2 of the manuscript.

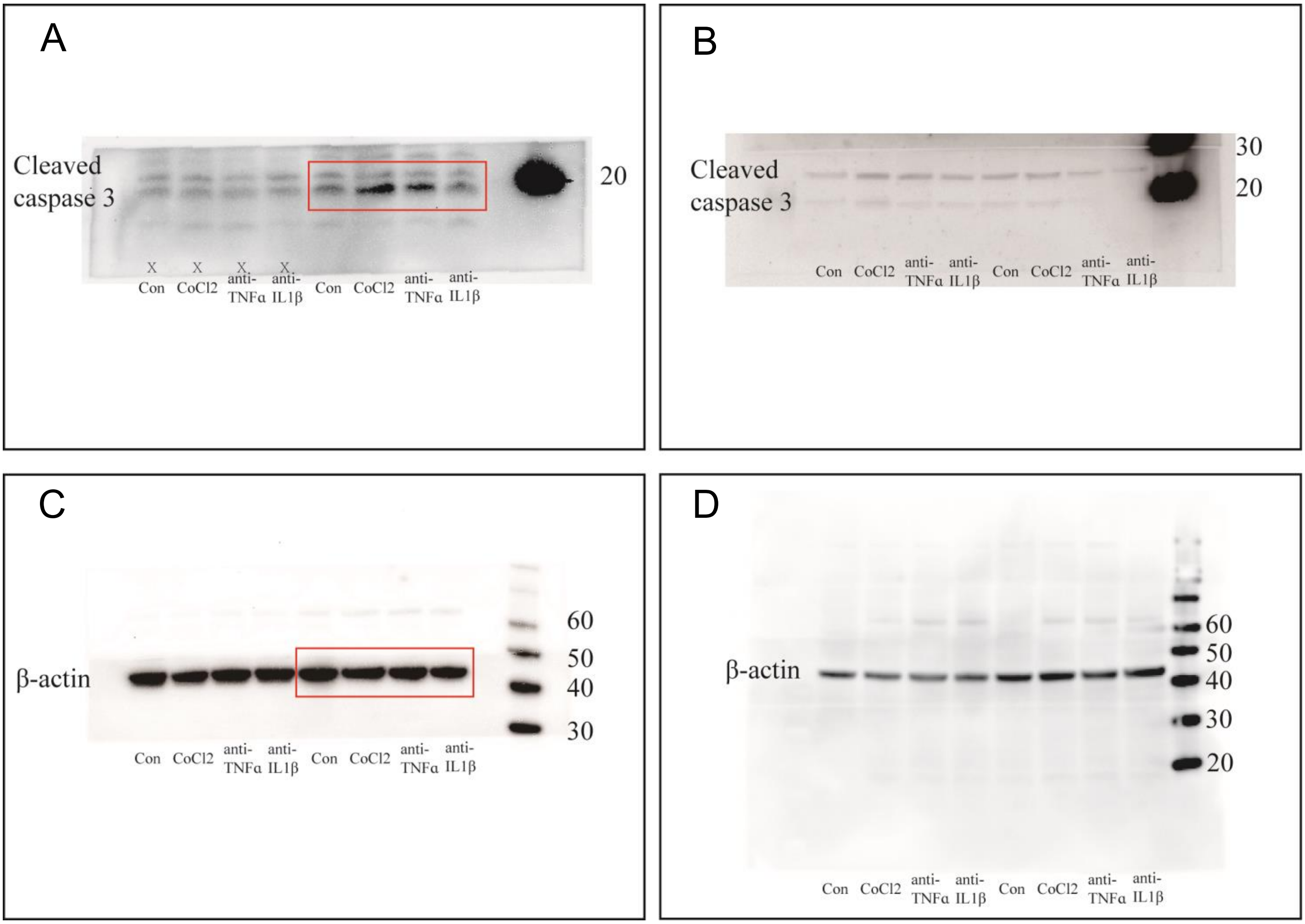

Figure S3. The entire uncropped images of the western blot for the protein cleaved-caspase 3 and the corresponding β-actin are shown in A – D, including four independent biological replicates. The bands marked by the red box in A and C are represented in the figure 3 of the manuscript.

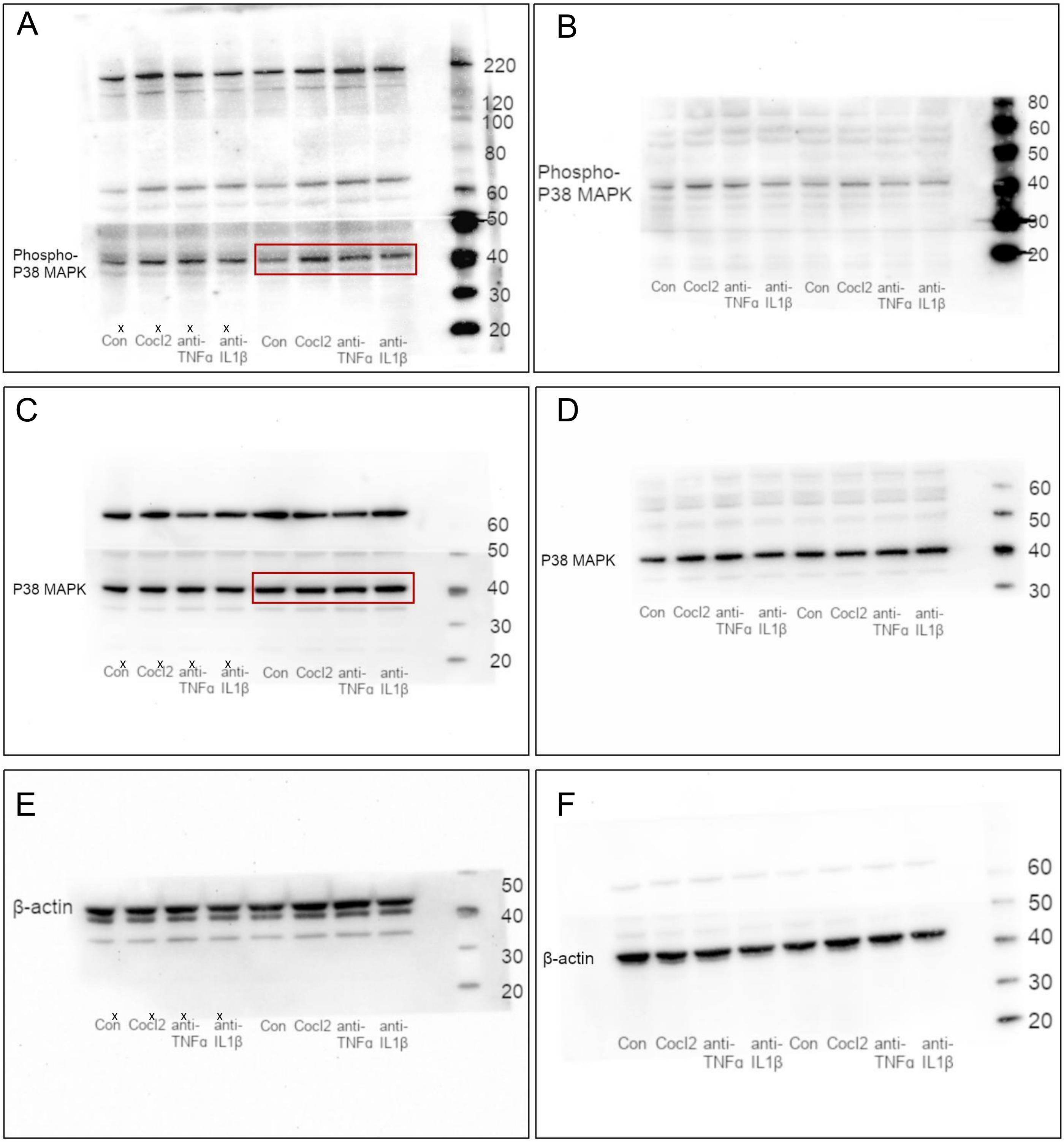

Figure S4. The entire uncropped images of the western blot for the protein phospho-p38 MAPK and the corresponding total p38 MAPK and β-actin are shown in A – F, including four independent biological replicates. The bands marked by the red box in A and C are represented in the figure 3 of the manuscript.

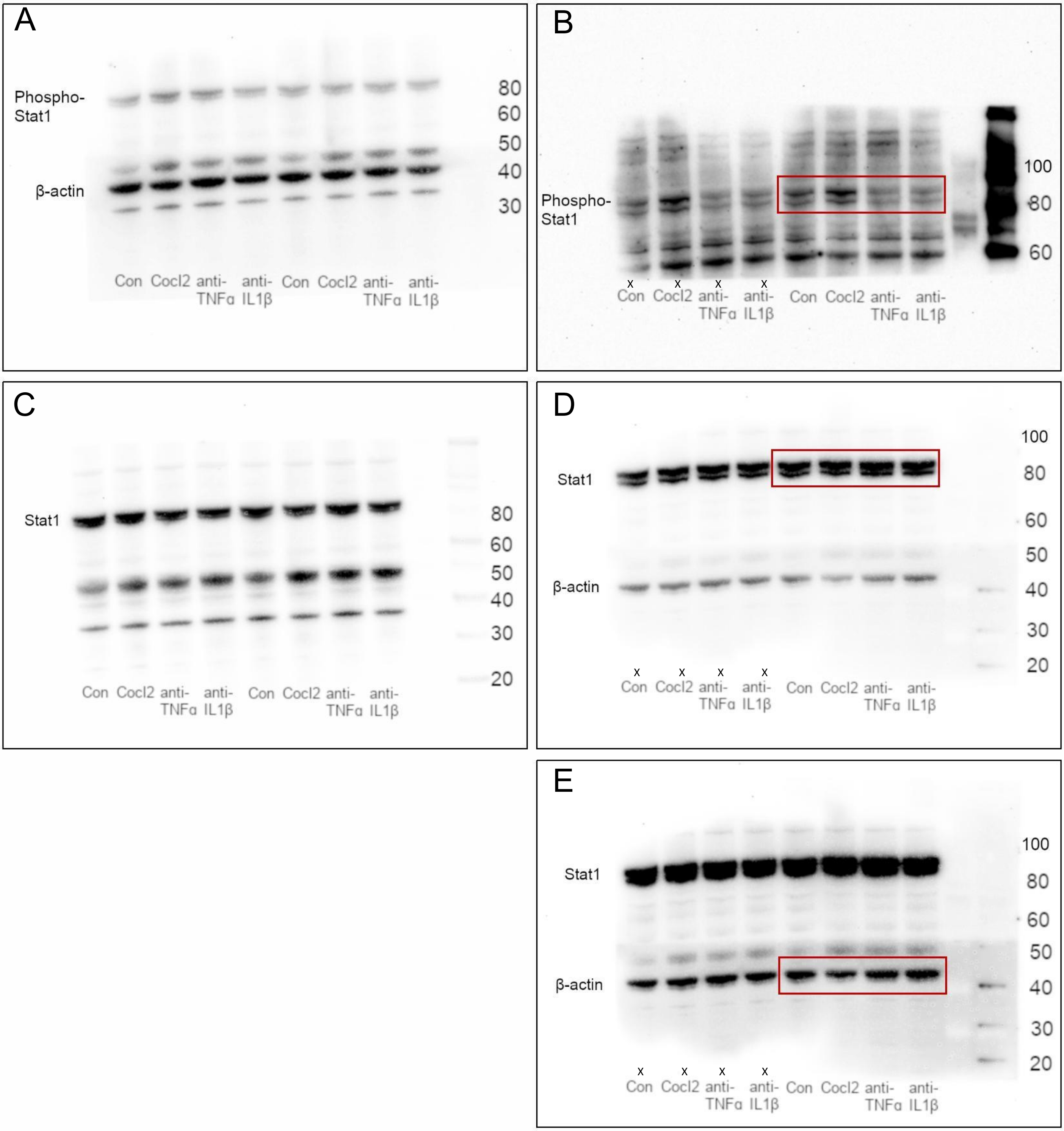

Figure S5. The entire uncropped images of the western blot for the protein phospho-STAT1 and the corresponding total STAT1 and β-actin are shown in A – E, including four independent biological replicates. The bands marked by the red box in A, D and E are represented in the figure 4 of the manuscript.

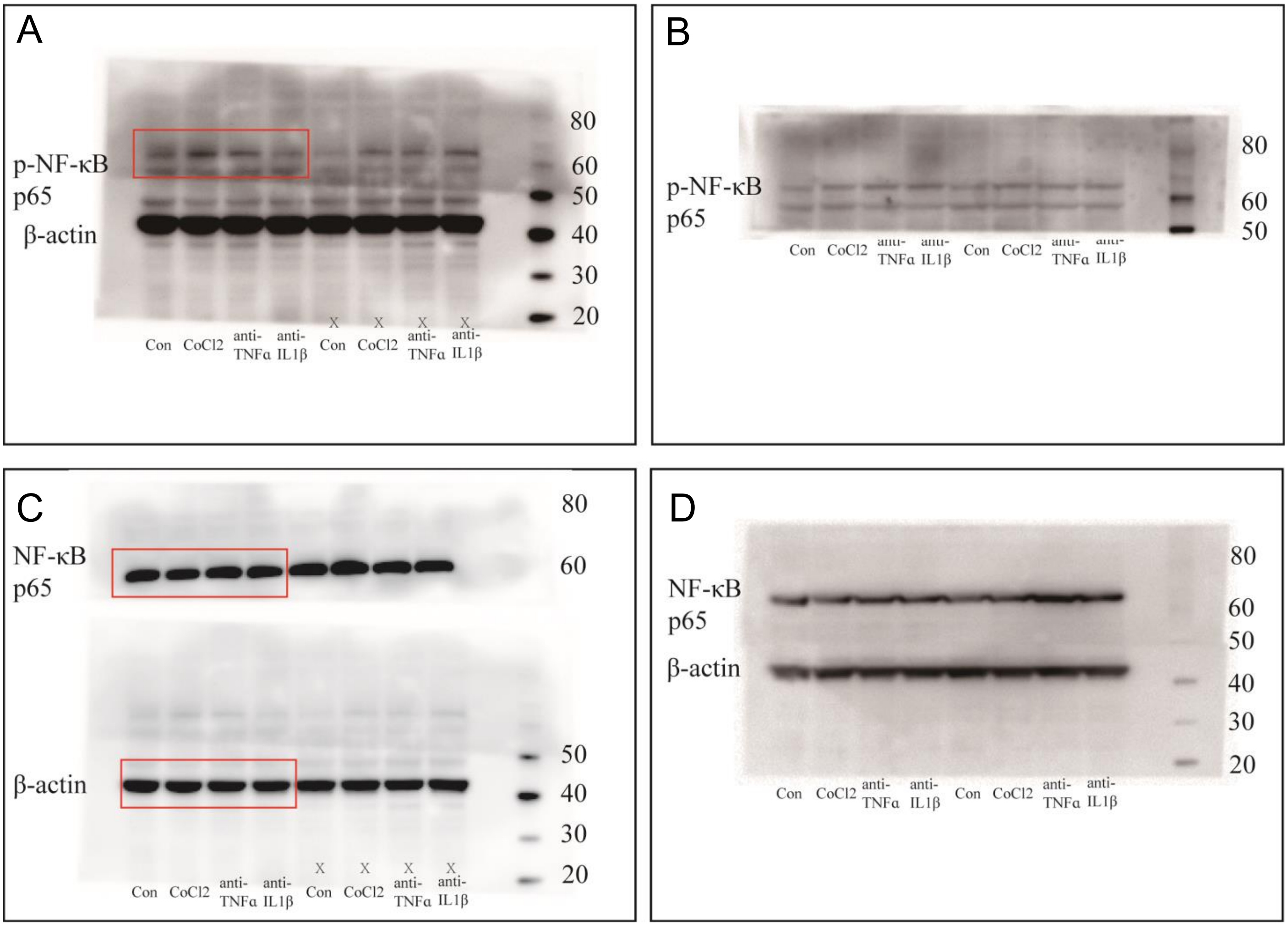

Figure S6. The entire uncropped images of the western blot for the protein phospho-NFκB p65 and the corresponding total NFκB p65 and β-actin are shown in A – D, including four independent biological replicates. The bands marked by the red box in A and C are represented in the figure 4 of the manuscript.

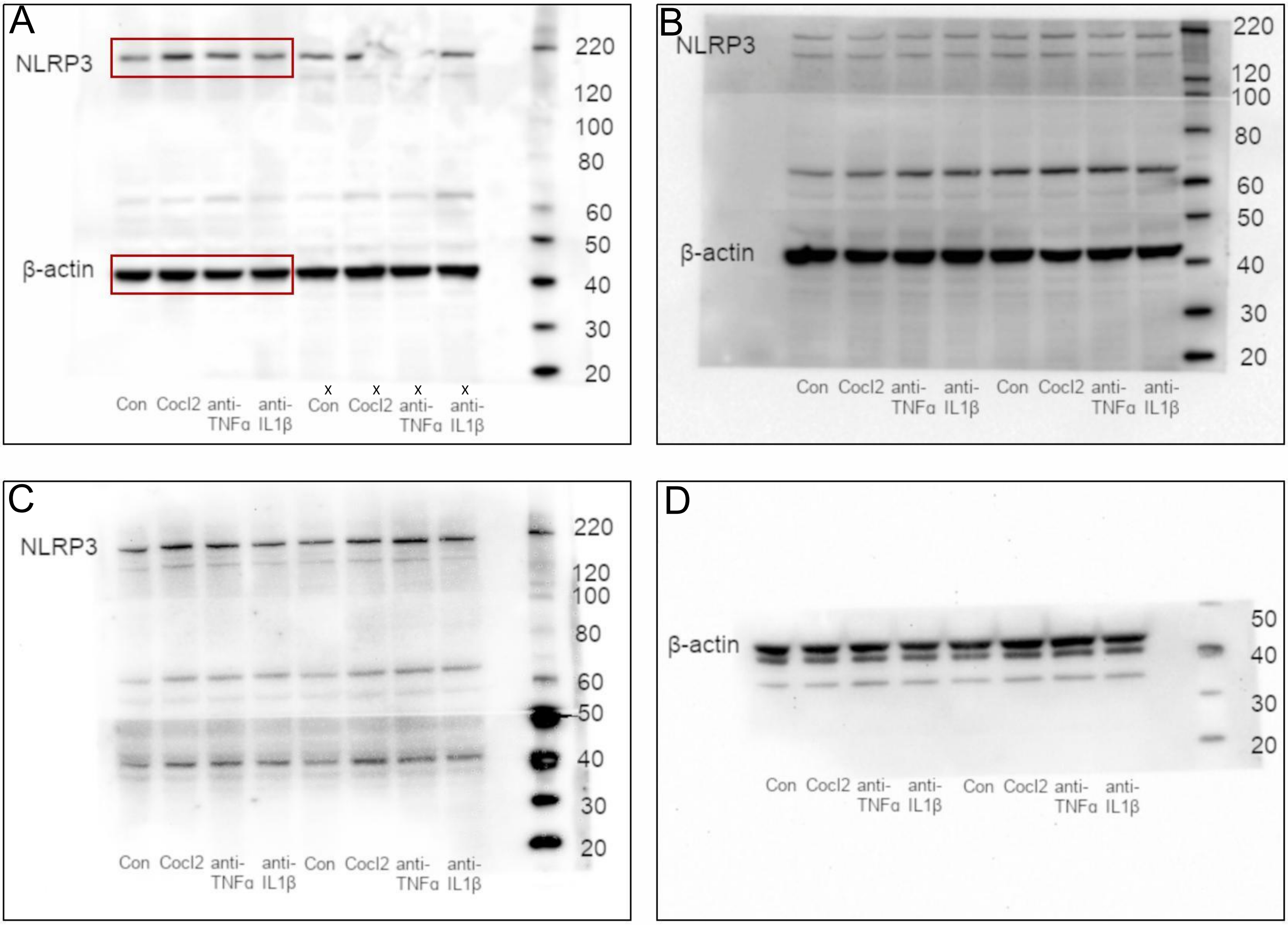

Figure S7. The entire uncropped images of the western blot for the protein NLRP3 and β-actin are shown in A – D (C and D were from the same gel), including five independent biological replicates. The bands marked by the red box in A are represented in the figure 4 of the manuscript.
